# Evolutionary binary feature selection using adaptive ebola optimization search algorithm for high-dimensional datasets

**DOI:** 10.1371/journal.pone.0282812

**Published:** 2023-03-17

**Authors:** Olaide N. Oyelade, Jeffrey O. Agushaka, Absalom E. Ezugwu

**Affiliations:** 1 Department of Computer Science, Faculty of Physical Sciences, Ahmadu Bello University, Zaria, Nigeria; 2 Unit for Data Science and Computing, North-West University, Potchefstroom, South Africa; Zonguldak Bülent Ecevit University: Zonguldak Bulent Ecevit Universitesi, TURKEY

## Abstract

Feature selection problem represents the field of study that requires approximate algorithms to identify discriminative and optimally combined features. The evaluation and suitability of these selected features are often analyzed using classifiers. These features are locked with data increasingly being generated from different sources such as social media, surveillance systems, network applications, and medical records. The high dimensionality of these datasets often impairs the quality of the optimal combination of these features selected. The use of the binary optimization method has been proposed in the literature to address this challenge. However, the underlying deficiency of the single binary optimizer is transferred to the quality of the features selected. Though hybrid methods have been proposed, most still suffer from the inherited design limitation of the single combined methods. To address this, we proposed a novel hybrid binary optimization capable of effectively selecting features from increasingly high-dimensional datasets. The approach used in this study designed a sub-population selective mechanism that dynamically assigns individuals to a 2-level optimization process. The level-1 method first mutates items in the population and then reassigns them to a level-2 optimizer. The selective mechanism determines what sub-population is assigned for the level-2 optimizer based on the exploration and exploitation phase of the level-1 optimizer. In addition, we designed nested transfer (NT) functions and investigated the influence of the function on the level-1 optimizer. The binary Ebola optimization search algorithm (BEOSA) is applied for the level-1 mutation, while the simulated annealing (SA) and firefly (FFA) algorithms are investigated for the level-2 optimizer. The outcome of these are the HBEOSA-SA and HBEOSA-FFA, which are then investigated on the NT, and their corresponding variants HBEOSA-SA-NT and HBEOSA-FFA-NT with no NT applied. The hybrid methods were experimentally tested over high-dimensional datasets to address the challenge of feature selection. A comparative analysis was done on the methods to obtain performance variability with the low-dimensional datasets. Results obtained for classification accuracy for large, medium, and small-scale datasets are 0.995 using HBEOSA-FFA, 0.967 using HBEOSA-FFA-NT, and 0.953 using HBEOSA-FFA, respectively. Fitness and cost values relative to large, medium, and small-scale datasets are 0.066 and 0.934 using HBEOSA-FFA, 0.068 and 0.932 using HBEOSA-FFA, with 0.222 and 0.970 using HBEOSA-SA-NT, respectively. Findings from the study indicate that the HBEOSA-SA, HBEOSA-FFA, HBEOSA-SA-NT and HBEOSA-FFA-NT outperformed the BEOSA.

## 1. Introduction

Recent technological advances have led to an increase in the amount of data generated and stored. The increase is in the volume and nature of the data, usually having large dimensions or features, outliers, skewness, missing values, redundant features, irrelevant data, integration, and heterogeneity [1, 2]. This increase significantly reduces the classifier’s accuracy, and the ability to manipulate this data decreases, too [3]. Hence the need for tools that can handle this volume of data. The issue of datasets with large dimensions and redundant or irrelevant features can be solved using the feature section methods [4]. These methods aim to reduce the number of features to the bearest minimum without information loss [5]. Feature selection (FS) methods have been successfully applied to many domains, including computational medicine [6, 7], clustering [8, 9], intrusion and spam detection [10–13], and genomics [14].

The methods for solving FS problems are broadly classified into filter-based, wrapper-based, and embedded-based methods. The filter-based methods reduce the number of features by assessing the features based on similarity, distance measure, information loss or gain, consistency, and statistical measures and then ranking these features based on these criteria [15]. The merit and demerit of the filter method are low computational cost and low performance, respectively. The wrapper-based methods perform feature reduction using a predetermined learning algorithm that evaluates all possible feature subsets to find the optimal one [16]. The wrapper has the advantage of providing higher classification accuracy than the others. Finally, the embedded methods are wrapper and filter-based hybrid methods. It has the advantages of filter-based and wrapper-based methods and incorporates the optimal feature search into the classifier training process [17].

Feature selection is an NP-hard problem because it involves finding an optimal subset out of 2^N^ subsets of a dataset with N features. Approximate algorithms such as metaheuristic algorithms have been used to find an optimal subset out of near-optimal subsets heuristically [18, 19]. Just like in other areas of application of metaheuristic algorithms, such as engineering problems [20, 21] and scheduling problems [22, 23], significant successes have been recorded in the area of FS [24, 25]. Emary et al. [26] used the wrapper-based method to propose two versions of binary grey wolf optimizer (bGWO) that use the stochastic crossover among the three best solutions and the S-shaped transfer function. The proposed methods were used to solve the FS problem. In the same two-way approach of converting the continuous search space to a binary one, Mafarja et al. [27] proposed a wrapper-based binary grasshopper optimization algorithm (BGOA) framework that uses the S-shaped and V-shaped transfer functions in the first instance and combines the finest solutions found so far. Their approach was used to solve the FS problem. The FS solution proposed by [28] is called an improved sine cosine algorithm (ISCA). It introduced an elitism technique and solution update mechanism that helps select an optimal feature subset and increases classification accuracy. The authors [29] used different variants of S-shaped and V-shaped transfer functions to develop eight binary variants of the newly proposed emperor penguin optimizer to solve the FS problem.

The dwarf mongoose optimization (DMO) proposed by [30] has been gaining attention from the metaheuristic research community. It was improved to a DMO-secure-based clustering and combined with a Multi-Hop Scheme Of Routing (DMOSC-MHRS) to solve the clustering problem [31]. The binary version of DMO (BDMO) was developed by [24] and was applied to solve the multiclass high-dimensional feature selection problem. The simulated annealing (SA) was used to improve the local search mechanism of the BDMO in the hybrid of the two algorithms and used to solve the FS problem [3]. The ebola optimization search algorithm (EOSA) [32] is a recently proposed swarm-based metaheuristic algorithm inspired by the Ebola virus disease propagation. It was used to solve 47 classical benchmark functions, and it significantly outperformed compared seven well-known state-of-the-art algorithms. The binary version of EOSA called BEOSA was proposed by [1] and used two newly formulated S-shape and V-shape transfer functions to investigate mutations of the infected population in the exploitation and exploration phases. The result of applying BEOSA on 22 benchmark datasets consisting of low, medium, and high dimensional data shows that the BEOSA and its variant BIEOSA significantly outperform the other known FS methods used for the comparisons.

Interestingly, the hybrids of these algorithms have proven to yield better performance in adequately solving real-life problems. For instance, in [33], authors hybridized Harris hawk optimization (HHO)–grey wolf optimization (GWO) to solve the challenge of ensuring unmanned aerial vehicles (UAV) avoid obstacles while releasing payload hold-release targets. Similarly, a study in [34] has demonstrated the use of a hybrid AI-based approach for solving real-life problems. Similarly, hybrid binary optimizers have also been proposed in the literature to address the feature selection problem. However, no study has investigated the complexity of integration/non-integration of both nested-transfer functions and the threshold method in solving this same problem. We consider that a hybrid of binary optimizers in investigating this design pattern in binary optimizer contributes to the current research focus in the domain. Moreover, this study is motivated by the applicability and feasibility of using nested-transfer functions and threshold methods to solve large high-dimensional datasets and compare performance with low-dimensional datasets. Furthermore, the study was also motivated to harness a recent binary optimizer (BEOSA) whose continuous variant has remained one of the state-of-the-art metaheuristic algorithms in leveraging its impressive optimization structures in investigating the approach proposed in this study.

This paper aims to overcome the curse of dimensionality difficulties in the FS domain by generating high-quality solutions. The hybrids are carefully aligned to improve the global and local searches to enhance the feature selection mechanism. Specifically, a novel hybrid binary optimization capable of effectively selecting features from increasingly high-dimensional datasets is proposed. The approach used in this study designed a sub-population selective mechanism that dynamically assigns individuals to a 2-level optimization process. The binary Ebola optimization search algorithm (BEOSA) is applied for the level-1 mutation, while the simulated annealing (SA) and firefly (FFA) algorithms are investigated for the level-2 optimizer. The level-1 method first mutates items in the population and then reassigns them to a level-2 optimizer. The selective mechanism determines what sub-population is assigned for the level-2 optimizer based on the exploration and exploitation phase of the level-1 optimizer. In addition, we designed nested transfer (NT) functions and investigated the influence of the function on the level-1 optimizer. The outcome of these are the HBEOSA-SA and HBEOSA-FFA, which are then investigated on the NT, and their corresponding variants HBEOSA-SA-NT and HBEOSA-FFA-NT with no NT applied. The hybrid methods were experimentally tested over high-dimensional datasets to address the challenge of feature selection. A comparative analysis was done on the methods to obtain performance variability with the low-dimensional datasets. The main contributions of this study are summarized as follows.

- This study introduced a novel hybrid binary optimization capable of effectively selecting features from increasingly high-dimensional datasets.
- The approach is based on a 2-level optimization process where a sub-population selective mechanism dynamically assigns individuals to the 2-level optimizer.
- The binary Ebola optimization search algorithm (BEOSA) is used as the level-1 mutation, while the simulated annealing (SA) and firefly (FFA) algorithms are used as the level-2 optimizer called HBEOSA-SA and HBEOSA-FFA, respectively.
- A novel nested transfer (NT) function is designed, and its influence on the level-1 optimizer is investigated, resulting in variants called HBEOSA-SA and HBEOSA-FFA.
- The hybrid methods were experimentally tested over high-dimensional datasets to address the challenge of feature selection.
- A comparative analysis was done on the methods to obtain performance variability with the low-dimensional datasets.

The rest of this manuscript is structured as follows: Related works are presented in Section 2. Section 3 discusses the methodology used in this study. The details about the datasets and performance metrics are presented in Section 4. Section 5 presents the experiments’ results and discusses this study’s findings. Finally, Section 6 provides the conclusion and possible future work.

## 2. Related works

There are many FS approaches in the literature that employs metaheuristics optimization methods [35]. In practice, the FS approach involves any of the Filter, Wrapper, Embedded, and Hybrid -approaches. The hybrid approach combines the best features of the Filter and Wrapper approaches to form one approach. Each of the Wrapper and Hybrid approaches have different ways of using metaheuristic algorithms for FS. The metaheuristic algorithm is adapted wholly the way it is or modified (improved to tackle FS peculiarities) or hybridized (combining best features of two or more metaheuristic algorithms). The terminologies hybrid and hybridize refer to different things. The hybrid refers to an FS approach, while hybridize refers to combining the best features of two or more metaheuristic algorithms.

This review starts with approaches that adapt or modify some metaheuristic algorithms for FS problems. Two novel binary algorithms based on the butterfly optimization algorithm (BOA) used the wrapper method to find the optimum features for efficiently classifying objects. The performance of the proposed approach was tested using over 21 datasets from the UCI repository and compared with four high-performance optimization algorithms [36]. Similarly, a dynamic butterfly optimization algorithm (DBOA) was proposed by enhancing the BOA using a local search algorithm based on mutation (LSAM). The enhancement prevents the BOA from being stuck in the local minima and is tested using 20 datasets found in the UCI repository. Their results show that DBOA outperforms candidate algorithms used in the study [37].

Different versions of the artificial butterfly optimization (ABO) were proposed by [38]. The first version is used for single-objective optimization, and the second and third are used for multi and many-objective FS optimization. The study was validated using 8 publicly available datasets, and their results showed the superiority of their proposed algorithms. An FS strategy using the particle swarm optimization (PSO) for improving the text clustering called (FSPSOTC) is proposed by [39]. They tested the performance of FSPSOTC using six regular text datasets characterized by an assortment of features. Their findings showed that FSPSOTC could assemble informative features by generating a subgroup of written descriptive features.

The authors [40] proposed a novel binary butterfly optimization algorithm for information gain (IG-bBOA) to solve the lack of redundancy and feature relevancy issue of the s-shaped binary butterfly optimization algorithm (S-bBOA). Six routine UCI registry datasets were used to test the proposed FS method’s performance. The results showed the superiority of the proposed method over other methods used for comparison. In [41], four text representation methods were used before the genetic algorithm (GA) was used to select the optimal set of features. The text representation methods used are the bag of words (BOW), N-gram, stemming, and conceptual representation.

Similar studies [42–44] used metaheuristic algorithms to find the optimal subset of features from text data found in three benchmark datasets. Specifically, invasive weed optimization (IWO) was used to find the optimal subset of features, and its accuracy was evaluated using the NB classifier. Their study was compared with PSO and GA [42]. In [43], all significant features are weighted using various Term Frequency (TF) methods consisting of TF, NORMTF, LOGTF, ITF, and SPARCK. The flower pollination algorithm (FPA) was then used to select the optimal set of features, and its accuracy was tested using the Ada-boost algorithm. Finally, in [44], the crow search algorithm (CSA) and KNN were used as an FS method and classifier, respectively.

Now the approaches that hybridized different metaheuristic algorithms are discussed. The goal is to create a robust method to select the relevant and optimum feature subset from the large feature sets in the original dataset. The authors [45] combined the best feature of the artificial bee colony (ABC) and bacterial foraging optimization (BFO) to form a wrapper-based hybrid called HABBFO. The hybridized HABBFO is then applied to select the most significant feature subset from Reuter’s dataset, which is later used for the prediction. The optimal feature subset is fed to an ANN, which performs the multi-label classification.

A three-step classification model was proposed by [46]. The author hybridized the grasshopper optimization algorithm (GOA) and crow search algorithm (CSA) to get a robust algorithm called (GCOA) used for the FS process. The vector space model (VSM) extracts features, and the Deep Belief Network (DBN) is used for text categorization (TC). Another hybridization of ant colony optimization (ACO) and GA called the ACOGA was proposed by [47]. The hybrid was used as an FS method and KNN as the classifier.

It is common knowledge that the major disadvantage of the wrapper-based FS approaches is the high cost of computational resources. The process of optimal feature subset identification is deeply embedded in the randomization mechanism of the algorithms. Many researchers have proposed a hybrid of intelligent optimization algorithms with traditional FS methods as a solution. This form of hybrid works by first performing preprocessing tasks that prune the data’s high dimension using any filter method. It then uses the wrapper-based metaheuristic method, which refines the selected feature subsets.

The authors [48] used the information gain (IG) and chi-square statistic (CHI) to preselect relevant feature subsets. Then, the preselected feature subset is further refined using a small-world optimization algorithm (SWA) to get the optimal feature subset. The KNN and SVM are used for text classification. In [49], the feature selection process is carried out in two phases. The filter method consisting of correlation (CO), information gain (IG), gain ratio (GR), and symmetrical uncertainty (SU) was used for preprocessing, while the wrapper-based PSO algorithm was used to refine the preselected feature subsets. The NB classifier was used to evaluate the optimally selected feature subset.

Likewise [50], proposed a hybrid FS method that used the Normalized Difference Measure (NDM) as a filter-based method and a wrapper-based Binary Jaya Optimization (BJO). The hybrid is called NDM-BJO and was used for the dimensionality reduction of feature space. The authors evaluated the selected feature subset using the NB and SVM. In [51], the Sine Cosine Algorithm (SCA) was improved and called (ISCA) for feature selection. However, the authors first used an information gain (IG) filter to rank the features and select the highest-ranked features, thereby reducing the size of high dimensionality. The NB algorithm was then used to validate the ISCA-selected feature subset.

The authors [52] modified the gaining sharing knowledge-based optimization algorithm (GSK) using the probability estimation operator called (Bi-GSK) to find the best feature subsets. The performance of Bi-GSK was enhanced using ten chaotic maps. The performance of these improved feature selection algorithms on twenty-one benchmark datasets taken from the UCI repository was compared with other existing algorithms, which showed that Chebyshev chaotic map has the best result among all chaotic. Similarly, the authors [53] used eight S-shaped and V-shaped transfer functions to binarize the GSK. The same datasets were used as previous authors, and the V4 transfer function outperforms other optimizers in terms of accuracy, fitness values, and the minimal number of features. The binary GSK has succeeded in other areas, such as the knapsack problem [54] and fault section location in distribution networks [55]. A decade-long survey of metaheuristic algorithms for feature selection (2009–2019) was presented in [56].

Undoubtedly, the use of metaheuristic algorithms for FS problems has been successful. However, it also comes with challenges, such as multi-objectivity, dynamicity, constraint, and uncertainty. Multi-objectivity implies multiple objectives that can be conflicting, and tradeoffs or Pareto optimal sets are needed for successful optimization. Uncertainty implies that the position of the global solution changes frequently. This scenario would require careful handling by these algorithms. The nature of the problem search space could lead to local minima stagnation and many more. The challenges of exploration and exploitation are enormous. They both serve conflicting purposes since increasing exploration may mean decreasing exploitation. Also, there is no clearly defined milestone for transiting between the two.

## 3. Methodology

The approach applied for the design of the proposed hybrid algorithms is presented in this section. First, the optimization process demonstrating how other algorithms are incorporated into the BEOSA method is presented. This model is further detailed using mathematical models. The design process also showed how each candidate solution is evaluated to obtain the best solution. Meanwhile, the transfer functions that support the binary optimizer are also detailed.

### 3.1 The hybrid HBEOSA model

The BEOSA [57] is a recent binary optimizer derived from the EOSA metaheuristics [58] and the immunity-based variant IEOSA [35]. The foundational design of the EOSA method was inspired by the Ebola virus and its associated propagation method. The base algorithm follows the susceptible, infected, recovered, exposed, hospitalized, vaccinated, quarantined, and death or dead (SIREHVQD) model. In this study, we leverage the EOSA and BEOSA to derive a new hybrid HBEOSA. The methodology follows a two-level (2-level) optimization approach using a novel nested transfer function. In this section, we describe the design of a level-1 optimizer using the BEOSA and then derive new methods using the integration of SA and FFA algorithms for the level-2 optimizer.

As inherited by the hybrid methods proposed in this study, an individual in the population initialization for the search space of BEOSAdy, is determined by Eq (1), while the entire population (*S*) of size *N*.


$${ind}_{i}=\sum_{j=0}^{sp}{ind}_{i,j}=1$$ (1)

$$individual=\left[\begin{array}{ccc} {ind}_{1,1} & \cdots & {ind}_{1,d}\\ \vdots & \ddots & \vdots \\ {ind}_{1,n} & \cdots & {ind}_{n,d} \end{array}\right]$$ (2)

where *sp* is *s*(*D*, *rnd*(*mn*, *mx*)); *mn* and *mx* which are representative of the 1 and ⌊0.5*D⌋ values; *D* is the dimension of each *ind*_*i*_ in the population; the *rnd*() returns a random positive non-zero integer value within the range of its parameter, and *S* is a sampling function that samples and returns a value within the range of [0, *D*].

An individual in *S* is positioned within a space and is allowed to move around to demonstrate the concept of infectiousness so that the individual can transit to the infected (*I*) compartment. As a result, position update for every *ind*_*i*_ in the system is computed using Eq (3).


$${mI}_{i}^{t+1}={mI}_{i}^{t}+rand(-1|0|1)*\rho$$ (3)

where ρ represents the scale factor of displacement of an individual, ${mI}_{i}^{t+1}$ and ${mI}_{i}^{t}$ are the updated and original position at time t and t+1, respectively. The *rand*(−1|0|1) randomly yields a value that can be -1 or 0, or 1, with each denoting movement leading to covered, intensification, and exposed displacements, respectively.

Only individuals exposed and infected are mutated, as represented using Eq (4). In the equation, the Δ notation denotes the change factor of an individual, *rand* represents a randomly generated uniform number in the range[−1, 1], *gbest* represents the current global best solution.


$${{ind}_{i}}^{new}=\Delta *e^{rand}cos(2\pi rand)*{(ind}_{i}-gbest)$$ (4)

#### 3.1.1 Simulated Annealing (SA)

The SA algorithm is considered the first method to hybridize with BEOSA for performance improvement. We take advantage of the core part of SA, which uses Eq (5) to update the current global best in the population by renaming *ind*_0_ with *ind*_*k*_ if Δ*f* returns a value less than zero; otherwise, we compute with Eq (6) and check if the condition *rand*<*p*(Δ*f*) is satisfied to confirm if *ind*_*k*_ still remains best global solution.


$$\Delta f=f({ind}_{k})-f({ind}_{0})$$ (5)

$$p(\Delta f)=e^{\left(\frac{\Delta f}{T}\right)}$$ (6)

#### 3.1.2 Firefly Algorithm (FFA)

The FFA, sometimes referred to as FA, is the second algorithm investigated for the hybridization process. The mutation of individuals in the algorithm is achieved using Eq (7).


$${ind}_{new}={ind}_{i}+\alpha *0.05urand+{2e}^{\left(-\gamma *r^{2}\right)}*({ind}_{j}*urand)$$ (7)

Where *r* is the radius and attraction level computed as $r=F({ind}_{i}-{ind}_{j})/\sqrt{D}$ and *F* is the Frobenius norm function; also *urand* represents a uniform random number in the range [0,1]; 0.05*urand* is computed as the mutation vector.

#### 3.1.3 Hybrid BEOSA (HBEOSA)

**Fig 1:**
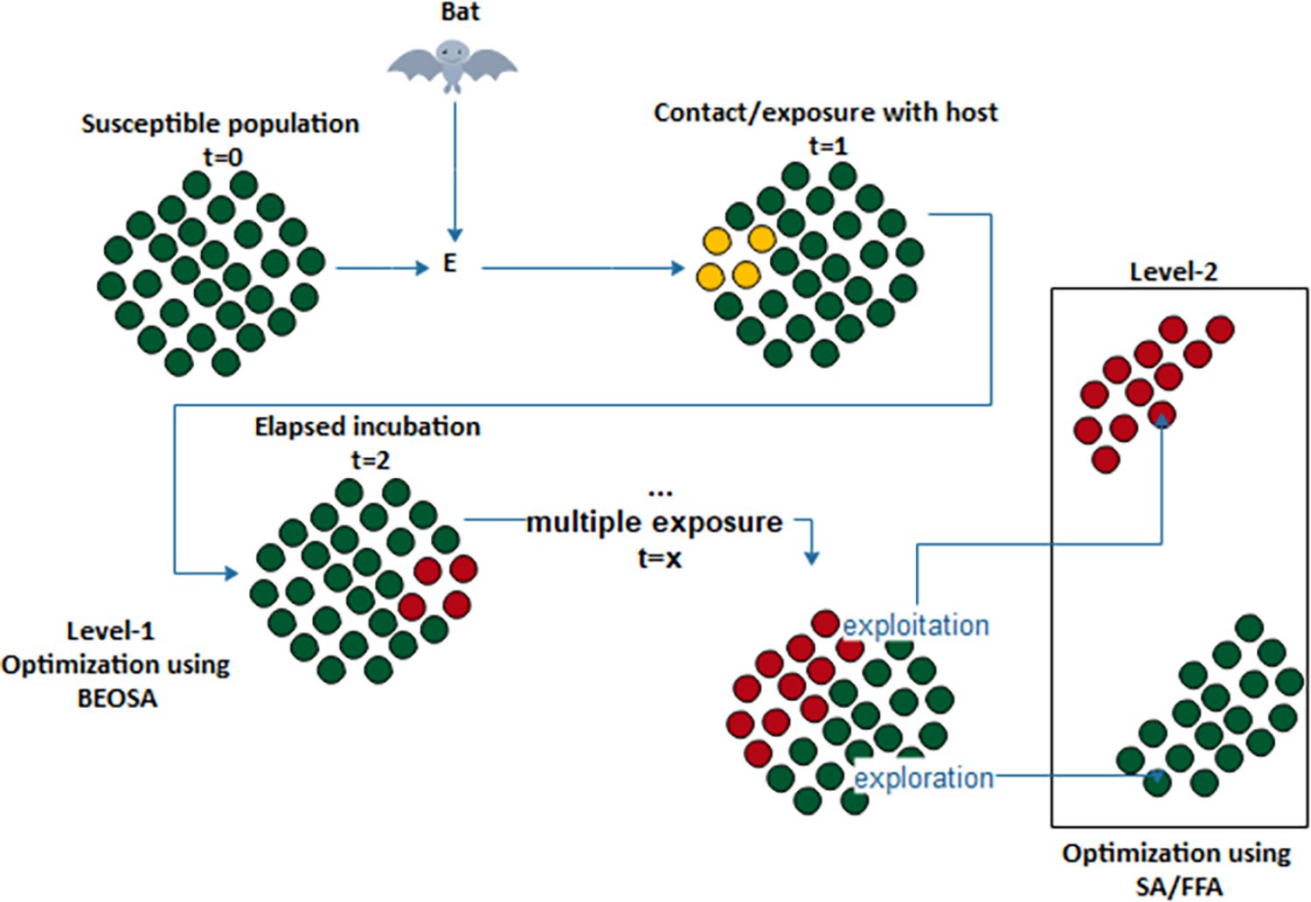
An optimization process of the proposed hybrid BEOSA (HBEOSA) combining both SA and FFA methods into BEOSA.

The optimization process described by the hybrid model is illustrated using Fig 1. We note that while the BEOSA initializes *S*, generates the number of infected to allocate to *Q*, and exposes a certain fraction of *S* to *I*, only during the infection stage is the integration of either SA or FFA applicable. Note that the hybrid allows for either individual in *S* to be further optimized with SA/FFA during the exploration phase of BEOSA, or we optimize the individuals of *I* using SA/FFA during the exploitation phase of BEOSA.

Therefore the hybrid model follows according to the mathematical models in Eq (8).


$$h(ind)=\{{\left(S\;I\right)}_{t}\to {optimize(ind}_{i})|{ind}_{i}\;\epsilon \;(S\;or\;I)\}$$ (8)

Where *h*(*ind*) represents the hybrid function which generates a set of individuals optimized by two methods with the BEOSA been the base method; the ⨁ and *optimize*() functions represent the BEOSA and SA/FFA optimization operators respectively, and *ind*_*i*_ is an element in the set of *S* or *I* at any time *t*.

The same fitness function is applied for evaluating solutions in the population used in BEOSA, SA, and FFA algorithms. This fitness function is described by Eq (9), which evaluates the solution based on its performance on a given classifier *clf* on a subset of the dataset $X[:1^{{ind}_{i}}]$ and the application of control parameter *ω*. The notation $1^{{ind}_{i}}$ as used in the equation, returns the number of 1s in the array representing the individual *ind*_*i*_. Note that the notation |*F*| returns the number of features selected in the individual while *D* represents the dimension of the features in the dataset *X*. For experimental purposes, the value of 0.99 was used for *ω* notation.


$$fit=\omega *(1-clf(X[:1^{{ind}_{i}}])+((1-\omega )\frac{\left|F\right|}{D})$$ (9)

Another evaluation function, known as the cost function, as described in Eq (10), was applied to check the cost-effectiveness of a potential solution. In contrast, the outcome from the previous equation demonstrates a solution’s fitness.


cost=1−fit (10)

In this study, we propose a novel approach to the design and use of transfer functions in binary optimization methods. The popular S, V, Z, and Q shapes have been reported and used in the literature. Nevertheless, we consider that a novel optimization and transformation outcome can be achieved using a nested transfer function. As a result, we modeled eight different transfer functions taking a cue from the basic S and V functions applied in our recent study [57]. In that previous study, we proposed using the S1 and S2 for the S-family and the V1 and V2 for the V-family transfer function. The first four transfer functions are categorized into the S-V function, while the other category is named the V-S function. In both categories, the nesting of the second term is achieved in the first term.

In equitation (11), we have the *S*1(*V*1) transfer function which first applied an arbitrary *ind*_*i*_ to *V*1 function, the outcome is then applied to the *S*1 function. A similar operation is designed for the *S*2(*V*1), *S*1(*V*2), and *S*2(*V*2) transfer functions, which are defined in Eqs (12–14).


S1(V1)=11+e(−(|x2+x2)|2) (11)

$$S2(V1)=1-\frac{1}{1+e^{\left(|\frac{x}{\sqrt{2+x^{2}}}|\right)}}$$ (12)

S1(V2)=11+e(−(|tanx|)2) (13)

$$S2(V2)=1-\frac{1}{1+e^{\left(|\tan\;x|\right)}}$$ (14)

Also, for the V-family, which is nested with the S-family, we show in Eqs (15–18) the definition for the *V*1(*S*1), *V*2(*S*1), *V*1(*S*2), and *V*2(*S*2), transfer functions.


V1(S1)=|(11+e(−x2))2+(11+e(−x2))2| (15)

V2(S1)=|tan(11+e(−x2))| (16)

$$V1(S2)=\left|\frac{\left(1-\frac{1}{1+e^{x}}\right)}{\sqrt{2+{\left(1-\frac{1}{1+e^{x}}\right)}^{2}}}\right|$$ (17)

$$V2(S2)=\left|\tan\left(1-\frac{1}{1+e^{x}}\right)\right|$$ (18)

**Fig 2:**
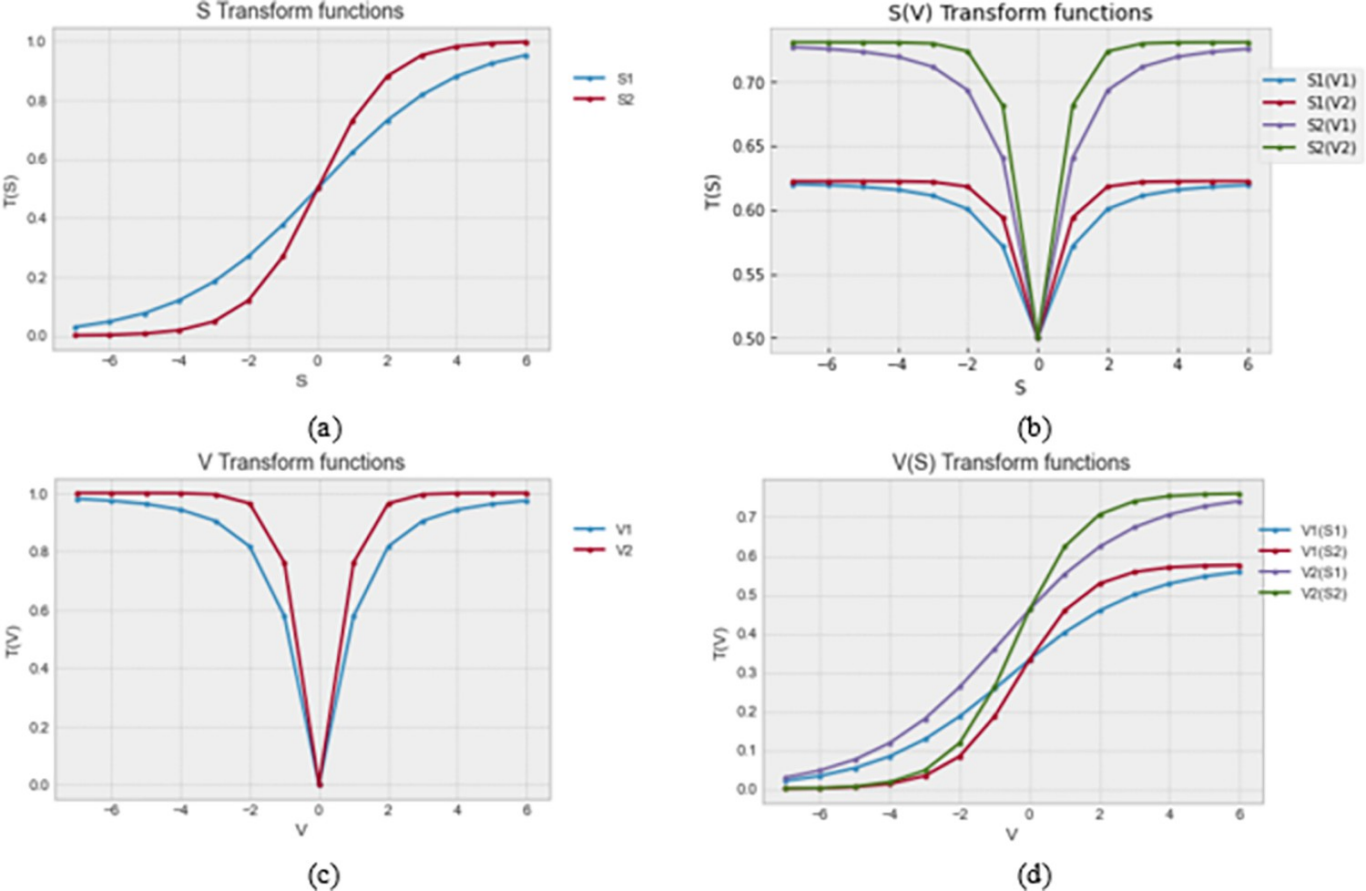
A graphical chart of the values of (a) two variants of *S* transfer functions (b) four variants of *S*(*T*) transfer functions, (c) two variants of *T* transfer functions, and (d) four variants of *T*(*S*) transfer functions.

Plotting the graph of the eight newly derived *SV*() transfer functions, we discovered an interesting shape that promises to impact the application process of the functions on solutions in the search space, thereby enhancing the optimization outcome. In Fig 2(A), we graphed the *S*1 and *S*2 transfer functions which form the basis of the four derived functions shown in Fig 2(B). While the original S-shaped transfer function illustrates that shape, the derived functions illustrate different versions of a V-shape with an inherent S-shape. We found this very interesting and suitable for testing the proposed hybrids of BEOSA. Similarly, the original V-shaped graphs consisting of *V*1 and *V*2 transfer functions are shown in Fig 2(C). The derived nested transfer functions resulting from this are displayed in Fig 2(D), and we see the shape of the plot curve according to the S-pattern, though having an inherent V-shape.

We demonstrate the applicability of the proposed nested derived transfer functions in the algorithm, which details the design of the hybrid algorithms.

### 3.2 Algorithmic and procedural flow of HBEOSA

The algorithmic design of the hybrid BEOSA methods is detailed in the sub-section with emphasis on the use of the transfer function as well as the branching from the BEOSA flow to the hybrids. In the algorithm, both SA and FFA methods are used for the hybrid to achieve what is referred to as HBEOSA-SA and HBEOSA-FFA. This study also investigates the possible performance of the hybrids when the derived transfer function is used and what the likely output would look like should the hybrids simply use a threshold approach with no transfer function. Hence, when the transfer functions are not used, new sets of hybrids, namely HBEOSA-SA-NT and HBOESA-FFA-NT, where the NT acronym defines non-transfer functions usage.

In Algorithm 1, the input and expected out for the hybrid algorithm are listed in Lines 1–2, while the body of the algorithm is listed in Lines 3–38. The initialization of the population and assignment of the index case of the infection on the population are described using Lines 4–5. Recall that the proposed method is designed to use the derived nested transfer functions and may not use the functions depending on the *isThreshold* control parameter assigned on Line 6. When the value for this parameter is set to true (1), the HBEOSA-SA and HBOESA-FFA algorithms are obtained, otherwise, we derive HBEOSA-SA-NT and HBOESA-FFA-NT from Algorithm 1.

**Algorithm 1. HBEOSA method.**

*1* ***Input*:** maxIter, psize, srate, lrate, dim

*2* ***Output***: gbest

*3* ***begin***

*4 S* = *Initialize and binarize populations (psize) as S*

5 *I*, *gbest*←*S*[0], *S*[0]

*6 isThreshold = rand*(1|0)

7 **while** *e < maxIter* ***and*** *size* (*I*) *>* 0 **do:**

*8 Compute individuals to be quarantine*

*9 I = difference of current infected cases (I) from quarantine cases*

10 **for** *i in 1 to size(I)* **do**:

11 generate new infected (nI) case from S

12 ${nI}_{i}=({nI}_{i}-gbest)*e^{rand}*\cos\left(2\pi *rand\right)$

13 **if**! *isThreshold*

14 **for** *j in 1 to dim* do:

15 *randomly generate* **d** between 1|0

16 **if** *displacement(nI*_*i*,_*) > 0*.*5* do:

17 *update size of nI using srate*

18 $s=\left\{ \begin{array}{l} S2(V1({nI}_{i,j}))|S2(V2({nI}_{i,j}))\;d=1\\ S1(V1({nI}_{i,j}))|S1(V2({nI}_{i,j}))\;d=0 \end{array}\right.$

19 **if** *s > = rand* do:

20 *nI*_*i*,*j*_ = 1

21 **else:**

22 *nI*_*i*,*j*_ = 0

23 **else:**

24 *update size of nI using lrate*

25 $v=\left\{ \begin{array}{l} V2(S1({nI}_{i,j}))|V2(S2({nI}_{i,j}))\;d=1\\ V1(S1({nI}_{i,j}))|V1(S2({nI}_{i,j}))\;d=0 \end{array}\right.$

26 **if** *t > = rand* do:

27 *nI*_*i*,*j*_ = 1

28 **else:**

29 *nI*_*i*,*j*_ = 0

30 if *displacement(nI*_*i*,_*)* < 0.5:

31 *nI* = SA(nI)|FFA(nI)

32 else:

33 *S* = SA(S)|FFA(S)

34 Evaluate new fitness of *nI*_*i*_

35 *I←nI*

36 *Update gbest and compartments variables*

37 ***return*** *gbest*

38 ***end***

The iterative process describing the optimization process is outlined in Lines 7–36, starting with the ***while*** structure, which has a conditional statement to terminate the loop. Further from this is the assignment of some infected (*I*) cases to the quarantine (*Q*) compartment, as shown in Lines 8–9. It is desired that every infected case has the potency to infect new cases from the susceptible *S* compartment, this is described with the first ***for*** loop structure and specifically model with Lines 11–12. The branching off from using the derived nested function is shown with Line 13 so that only the design of HBEOSA-SA and HBEOSA-FFA is seen listed between Lines 14–29. The use of the transfer function in the case of the exploration and exploitation phases of the algorithm is shown in Lines 18 and 25, respectively. The return value for *s* and *t* and as conditioned with a randomly generated number, determines if a 1 or 0 is assigned to *nI*_*i*,*j*_ element of the infected case being transformed. Note the use of SA and FFA to optimize *nI* and *S* on Lines 31 and 33. This demonstrates that only when the algorithm is in the exploitation phase is either SA or FFA applied to optimize individuals in the newly infected *nI* compartments; otherwise, the entire population remaining in *S* is optimized.

**Fig 3:**
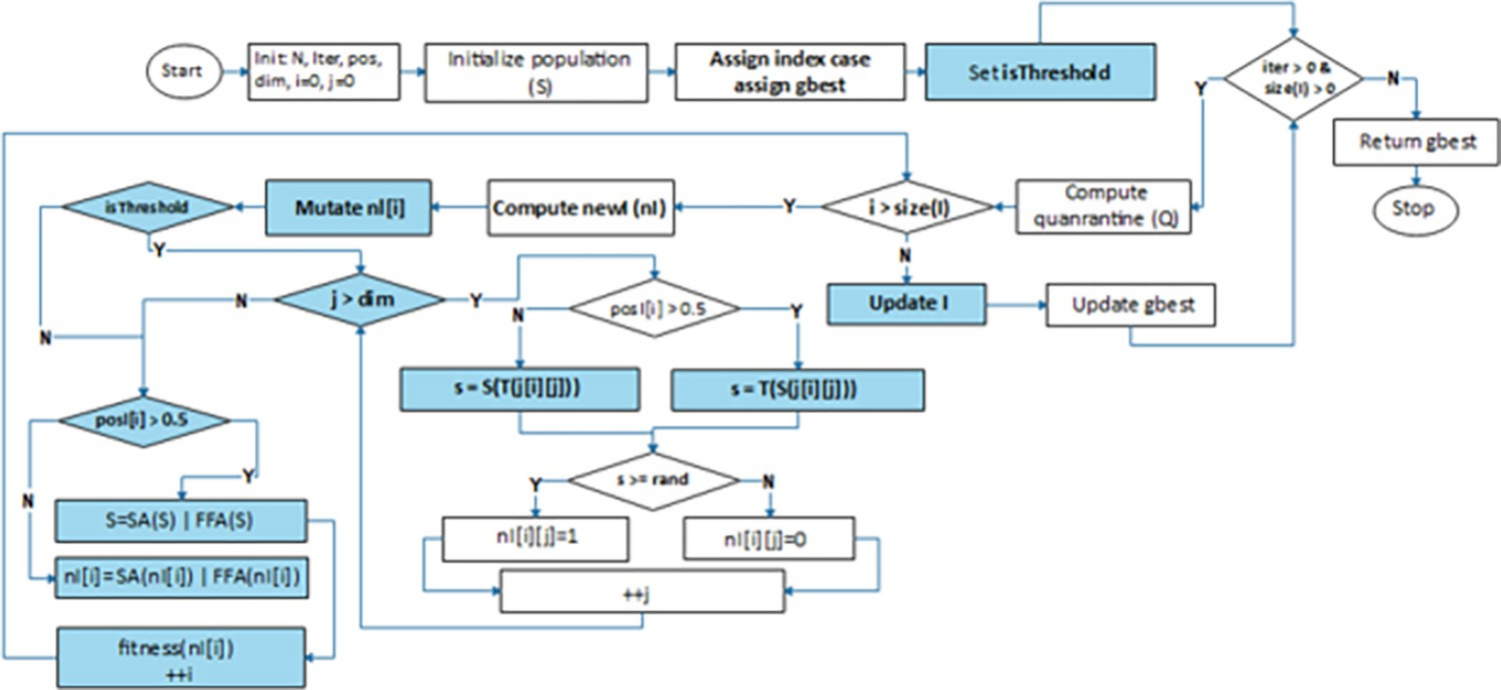
The flowchart showing the optimization process of the hybrid methods using BEOSA as the base algorithm and SA and FFA as integrated algorithms.

The flowchart further detailing the data flow within the algorithm described above is shown in Fig 3. We differentiate the flowchart of HBEOSA from that of BEOSA using some colored boxes. The highlighted boxes showed the use of the *isThreshold* control parameter, the mutation of the newly infected case *nI*_*i*_. Once the mutation operation is applied, the checking for the use of the *isThreshold* parameter is testing to determine the branching of the flowchart either to run HBEOSA-SA and HBEOSA-FFA or HBEOSA-SA-NT and HBEOSA-FFA-NT. Note also the highlight of the boxes showing the derived transfer functions and the use of the SA/FFA methods for further optimization.

The method described in this section demonstrates the proposed hybrid BEOSA (HBEOSA) algorithms. This hybrid algorithm is further used to derive two methods, the HBEOSA-SA and the HBEOSA-FFA. Furthermore, we showed the design of a novel transfer function and mentioned that the applicability of the functions to the solving of feature selection problem is tested using two variants of the proposed hybrids, namely the HBEOSA-SA-NT and HBEOSA-FFA-NT. The following sections discuss the detailing of the datasets, experimentation, evaluation criteria, results, and discussion of the proposed method.

## 4. Datasets and evaluation metrics

The performance of the hybrids of the BEOSA algorithm is evaluated using publicly available datasets, which can be categorized into high-dimensional, medium-dimensional, and low-dimensional [59]. The high-dimensional datasets include WaveformEW, Sonar, PenglungEW, KrVsKpEW, Ionosphere, BreastEW, Prostate, colon, and Leukemia. Those categorized in the medium-scale group include the Zoo, Vote, SpectEW, Lymphography, and CongressEW. The Iris, Wine, Tic-tac-toe, M-of-n, HeartEW, Exactly, Exactly2, and BreastCancer are grouped into the low-dimensional dataset. Details about the datasets used for the experimentation in this study are given in Table 1.

**Table 1:** Datasets and their properties.

Dataset	Number of Features	Number of Instances	Number of Class	Description
Leukemia	7070	72	2	Biology-based and medical-oriented dataset
Prostate	5966	102	2	Biology-based and medical-oriented dataset
WaveformEW	5000	40	3	sampled at 21 intervals. Each class is a random convex combining 2 out of 3 base waves.
Lung	3312	203	5	Biology-based and medical-oriented dataset
KrVsKpEW	3196	36	2	Game dataset A generator dataset generates three classes of waves, with each class
Colon	2000	62	2	Biology-based and medical-oriented dataset
Exactly	1000	13	2	Artificial binary classification dataset
Exactly2	1000	13	2	Artificial binary classification dataset
M-of-n	1000	13	2	Biology-based and medical-oriented dataset
Tic-tac-toe	958	9	2	Endgame dataset
BreastEW	569	30	2	Biology-based and medical-oriented dataset
CongressEW	435	16	2	Congressional voting dataset
Ionosphere	351	34	2	Electromagnetic dataset
PenglungEW	325	73	7	Biology-based and medical-oriented dataset
Vote	300	16	2	Electioneering domain
HeartEW	270	13	2	Biology-based and medical-oriented dataset
SpectEW	267	22	2	Biology-based and medical-oriented dataset
Sonar	208	60	2	Sonar signal classification dataset
Wine	178	13	3	Wine dataset showing the results of analysis of chemicals in wines.
Lymphography	148	18	4	Biology-based and medical-oriented dataset
Zoo	101	16	7	Biology-based dataset
Iris	4	150	2	Biology-based dataset

The experiments in this study were conducted using a personal computer (PC) with the following configuration: CPU, Intel® Core i5-4210U CPU 1.70 GHz, 2.40 GHz; RAM of 8 GB; Windows 10 OS. This was complemented with other computer systems having Intel® Core i5-4200, CPU 1.70 GHz, 2.40 GHz; RAM of 16 GB; 64-bit Windows 10 OS. The hybrid metaheuristic algorithms were implemented using Python 3.7.3 and supporting libraries, such as Numpy and other dependent libraries.

The comparative performances of all hybrid methods were considered under the following measures: classification accuracy, cost and fitness function values, the number of features selected, and computational time. Tabular and graph-based result outlines were shown based on the HBEOSA-SA, HBEOSA-SA-NT, HBEOSA-FFA, HBEOSA-FFA-NT, and BEOSA algorithms. Furthermore, to discover the performance of each algorithm concerning population variation, we subjected the experimentation to 50 and 100 population sizes for every run using 50 iterations. Table 2 presents the parameter settings of the base algorithm (BEOSA) used to derive the hybrids (HBEOSA-SA, HBEOSA-SA-NT, HBEOSA-FFA, HBEOSA-FFA-NT).

**Table 2:** Parameter settings.

Method	Parameter	Value	Definition
BEOSA	n	0.1	Recruitment rate
	p1, p2, p3 and p4	0.1, 0.1, 0.1, and 0.1	Contact rate of infected individuals, of the host, with the dead, and with the recovered individuals

## 5. Results and discussion

The result of the experimentation carried out in the study are presented in this section. Emphasis is made on a comparative approach in the presentation of the outcome. As a result, the comparative performances of all hybrid methods were considered under the following measures: the classification accuracy, the cost and fitness function values, the number of features selected, and, lastly, the computational time. Tabular and graph-based result outlines were shown based on the HBEOSA-SA, HBEOSA-SA-NT, HBEOSA-FFA, HBEOSA-FFA-NT, and BEOSA algorithms. This is motivated by the need to observe the performance of proposed hybrids of BEOSA to allow for an investigative reportage of these performances and suitability for practical applicability. Furthermore, to discover the performance of each algorithm with respect to population variation, we subjected the experimentation to both 50 and 100 population sizes for every run using 50 iterations. The section concludes by highlighting the study findings based on the metrics supporting the feature selection process.

The investigation of the hybrids of the BEOSA algorithm is considered under the categorization of the datasets into high-dimensional, medium-dimensional, and low-dimensional. The high-dimensional datasets include WaveformEW, Sonar, PenglungEW, KrVsKpEW, Ionosphere, BreastEW, Prostate, colon, and Leukemia. Those categorized in the medium-scale group include the Zoo, Vote, SpectEW, Lymphography, and CongressEW. The Iris, Wine, Tic-tac-toe, M-of-n, HeartEW, Exactly, Exactly2, and BreastCancer are grouped into the low-dimensional dataset.

### 5.1 Comparative analysis of features count by hybrid methods

The evaluation of the number of features selected by HBEOSA-SA, HBEOSA-SA-NT, HBEOSA-FFA, HBEOSA-FFA-NT, and BEOSA algorithms are discussed in the sub-section. Considering that the study aims to observe the most performing method based on the number of features selected, we highlight methods with the most optimal number of features and mention those with the worst performance in terms of selected features. Table 3 shows a comparative listing of the feature counts reported by all datasets in the category of high-dimensional scale. Some algorithms, such as HBEOSA-FFA and HBEOSA-SA, underperformed by returning a negligent number of features in almost all the datasets in the category. This is reflected in the table by those rows with a zero (0) value as the corresponding values for the feature count column. The implication of this as regards the HBEOSA-FFA and HBEOSA-SA algorithms on those datasets is the issue of the unsuitability of the method due to the integration of transfer function in their design. On the other hand, the same methods, HBEOSA-FFA and HBEOSA-SA, which were not designed with transfer functions, performed well for all datasets in the category of high-dimensional scale.

**Table 3:** Large-scale dataset comparative analysis using the number of features selected.

Dataset	Algorithm	Feature Count	Dataset	Algorithm	Feature Count
**BreastEW**	HBEOSA-SA-NT	11.2	**Prostate**	HBEOSA-SA	0
	HBEOSA-FFA	0		HBEOSA-SA-NT	530
	HBEOSA-FFA-NT	8.2		HBEOSA-FFA	0
	BEOSA	5		HBEOSA-FFA-NT	441.4
**Colon**	HBEOSA-SA	0	**Leukemia**	HBEOSA-SA	0
	HBEOSA-SA-NT	646.4		HBEOSA-SA-NT	737.2
	HBEOSA-FFA	0		HBEOSA-FFA	0
	HBEOSA-FFA-NT	168.4		HBEOSA-FFA-NT	109.2
	BEOSA	0		BEOSA	74
**Ionosphere**	HBEOSA-SA	0	**PenglungEW**	HBEOSA-SA	0
	HBEOSA-SA-NT	9		HBEOSA-SA-NT	61.5
	HBEOSA-FFA	0		HBEOSA-FFA	0
	HBEOSA-FFA-NT	3		HBEOSA-FFA-NT	101.5
	BEOSA	2		BEOSA	9.732358
**KrVsKpEW**	HBEOSA-SA	0	**Sonar**	HBEOSA-SA	0
	HBEOSA-SA-NT	18.5		HBEOSA-SA-NT	23
	HBEOSA-FFA	0		HBEOSA-FFA	0
	HBEOSA-FFA-NT	21		HBEOSA-FFA-NT	19.5
	BEOSA	12.89856		BEOSA	25
**WaveformEW**	HBEOSA-SA	0			
	HBEOSA-SA-NT	17			
	HBEOSA-FFA	0			
	HBEOSA-FFA-NT	18			
	BEOSA	20			

The performances of HBEOSA-FFA-NT and HBEOSA-SA-NT on WaveformEW, Sonar, PenglungEW, KrVsKpEW, Ionosphere, BreastEW, Prostate, colon, and Leukemia returned different results, which are worth considering. HBEOSA-FFA-NT performed better than HBEOSA-SA-NT though BEOSA outperformed all methods on the BreastEW dataset. A similar performance is observed for Prostate, Leukemia, Ionosphere, and KrVsKpEW. With regards to the Colon dataset, almost a similar report is obtained except for the inadequacy of BEOSA to compete with its hybrids. The HBEOSA-SA–NT did well with the WaveformEW dataset, followed by HBEOSA-FFA-NT and BEOSA in that order. The result on the PenglungEW dataset showed that while HBEOSA-SA–NT still leads, BEOSA comes out better than HBEOSA-FFA–NT, which lags far behind in performance. The summary of all these performances reveals that HBEOSA-SA–NT and HBEOSA-FFA–NT are competitive, outperforming the basic BEOSA, and are much more applicable for extracting the optimal combination of features needed for the classification purpose. Therefore, this showed that the use of transfer function in binary optimization method is not as significant as reported in the literature. Nevertheless, a careful hybridization of binary optimizers could yield better performance for a high-dimensional dataset.

The results obtained for the medium-dimension dataset are listed in Table 4 where the following are considered: Zoo, Vote, SpectEW, Lymphography, and CongressEW. Similar to the observation noted for the high-dimensional dataset, we see that both the HBEOSA-SA and HBEOSA-FFA showed that their performance was impaired due to the use of the transfer function, the CongressEW is an exception to this observation. On the contrary, their corresponding methods, HBEOSA-SA-NT and HBEOSA-FFA-NT which did not use the transfer function, returned a good number of features selected. In all the datasets, we found BEOSA returning suboptimal feature counts compared with its hybrids of HBEOSA-SA-NT and HBEOSA-FFA-NT. For instance, HBEOSA-SA-NT showed the best performance with the CongressEW dataset, while HBEOSA-FFA-NT demonstrated this same superiority with Vote, Zoo, and Lymphography. The implication is that a hybrid of BEOSA and FFA algorithm is much more compatible and productive when compared with the hybrid of BEOSA and SA.

**Table 4:** Medium-scale dataset comparative analysis using the number of features selected.

Dataset	Algorithm	Feature Count	Dataset	Algorithm	Feature Count
**CongressEW**	HBEOSA-SA	7.5	**Vote**	HBEOSA-SA	0
	HBEOSA-SA-NT	4.5		HBEOSA-SA-NT	5.5
	HBEOSA-FFA	0		HBEOSA-FFA	0
	HBEOSA-FFA-NT	5		HBEOSA-FFA-NT	4.5
	BEOSA	5		BEOSA	3.415831
**Lymphography**	HBEOSA-SA	0	**Zoo**	HBEOSA-SA	0
	HBEOSA-SA-NT	7		HBEOSA-SA-NT	6.5
	HBEOSA-FFA	0		HBEOSA-FFA	0
	HBEOSA-FFA-NT	4.5		HBEOSA-FFA-NT	5
	BEOSA	6.524479		BEOSA	8
**SpectEW**	HBEOSA-SA	0			
	HBEOSA-SA-NT	9.5			
	HBEOSA-FFA	0			
	HBEOSA-FFA-NT	12			
	BEOSA	7			

Six datasets were experimented with under low-dimensional datasets, and the results obtained are listed in Table 5. HBEOSA-SA-NT and HBEOSA-FFA-NT are seen to compete closely here, especially with the Iris and Exactly datasets. However, the former seems to outperform the latter in M-of-n, Tic-tac-toe, and Exactly2 datasets while lagging in the Wine dataset. This again confirms that HBEOSA-FFA-NT remains the best hybrid of BEOSA to yield optimal performance in terms of the number of features selected for classification purposes. Recall that we had observed this performance trend with high-dimensional, medium-dimensional, and now low-dimensional datasets.

**Table 5:** Small-scale dataset comparative analysis using the number of features selected.

Dataset	Algorithm	Feature Count	Dataset	Algorithm	Feature Count
**Exactly**	HBEOSA-SA	0	**M-of-n**	HBEOSA-SA	0
	HBEOSA-SA-NT	4.5		HBEOSA-SA-NT	6
	HBEOSA-FFA	0		HBEOSA-FFA	0
	HBEOSA-FFA-NT	4.5		HBEOSA-FFA-NT	5
	BEOSA	2.335506		BEOSA	6
**Exactly2**	HBEOSA-SA	0	**Tic-tac-toe**	HBEOSA-SA	0
	HBEOSA-SA-NT	6.5		HBEOSA-SA-NT	6
	HBEOSA-FFA	0		HBEOSA-FFA	0
	HBEOSA-FFA-NT	4		HBEOSA-FFA-NT	4.5
	BEOSA	5		BEOSA	5
**Iris**	HBEOSA-SA	0	**Wine**	HBEOSA-SA	0
	HBEOSA-SA-NT	1.2		HBEOSA-SA-NT	3
	HBEOSA-FFA	0		HBEOSA-FFA	0
	HBEOSA-FFA-NT	1.2		HBEOSA-FFA-NT	4.5
	BEOSA	1		BEOSA	4

The summary of the methods’ performance compared with the number of features selected on all categories of the dataset is that applying hybrid algorithms is more suitable. Furthermore, we observed that using a transfer function could greatly impair the hybrid methods’ performance when such a function’s design and integration are ineffective. We also noted that FFA’s hybrids with BEOSA yield better performance than the hybrid with SA. This shows that the biology-swarm-based nature of BEOSA and FFA might be the reason for the good performance reported by the hybrid. Recall those swarm-based algorithms are often more competitive than those physics-based.

### 5.2 Comparative analysis of classification accuracy by hybrid methods

The problem of feature selection is evaluated by investigating the outcome of the classification accuracy resulting from using the selected features. In this study, we experimented with the features selected by HBEOSA-SA, HBEOSA-SA-NT, HBEOSA-FFA, HBEOSA-FFA-NT, and BEOSA. Further, we investigated the impact of varying the population size for each method, and an average classification accuracy value was computed. Results are presented and compared in the three categories of datasets followed in the last sub-section.

Table 6 lists the result obtained for the high-dimensional datasets, including the WaveformEW, Sonar, PenglungEW, KrVsKpEW, Ionosphere, BreastEW, Prostate, colon, and Leukemia. In most cases, we found that only minimal differences existed between the classification accuracy obtained for experiments using a population size of 50 and those using a population size of 100. This is readily noticeable with the BreastEW and Colon datasets. The analysis aims to see the impact of the reduced features in achieving good classification accuracy. It is desired that such accuracy must be significant; otherwise, we conclude that the feature selected is suboptimal. Hence the binary optimizers proposed are ineffective. As an example, we note that the average classification accuracy observed for HBEOSA-SA, HBEOSA-SA-NT, HBEOSA-FFA, HBEOSA-FFA-NT, and BEOSA for BreastEW, Colon, Ionosphere, KrVsKpEW, Leukemia, and Prostate range between [0.91–1.00] except for HBEOSA-SA on Ionosphere which yielded 0.867857, though the general classification outcome that is very significant and appreciable. On the other hand, we see the classification accuracy reported for PenglungEW, Sonar, and WaveformEW with the same algorithms, namely HBEOSA-SA, HBEOSA-SA-NT, HBEOSA-FFA, HBEOSA-FFA-NT, and BEOSA, had their classification lower, though significant, in the range of [0.7166–0.8809]. This result implies that all the hybrids of BEOSA as proposed in the study, are suitable for fetching only relevant features required for obtaining significant classification accuracy, even on high-dimensional datasets.

**Table 6:** Large-scale dataset comparative analysis using classification accuracy for population sizes 50 and 100.

Dataset	Algorithm	Acc50	Acc100	Avg. Acc.
**BreastEW**	HBEOSA-SA-NT	0.935088	0.921053	0.92807
	HBEOSA-FFA	0.959649	0.929825	0.944737
	HBEOSA-FFA-NT	0.966667	0.933333	0.95
	BEOSA	0.947368	0.947368	0.947368
**Colon**	HBEOSA-SA	1	1	1
	HBEOSA-SA-NT	0.953846	0.984615	0.969231
	HBEOSA-FFA	1	1	1
	HBEOSA-FFA-NT	1	1	1
	BEOSA	1	1	1
**Ionosphere**	HBEOSA-SA	0.871429	0.864286	0.867857
	HBEOSA-SA-NT	0.935714	0	0.917857
	HBEOSA-FFA	0.842857	0.8	0.846429
	HBEOSA-FFA-NT	0.928571	0.914286	0.921429
	BEOSA	0.942857	0.942857	0.942857
**KrVsKpEW**	HBEOSA-SA	0.953052	0.893584	0.923318
	HBEOSA-SA-NT	0.92097	0.960094	0.940532
	HBEOSA-FFA	0.953052	0.943662	0.948357
	HBEOSA-FFA-NT	0.932707	0.945227	0.938967
	BEOSA	0.948357	0.948357	0.948357
**Leukemia**	HBEOSA-SA	0.973333	0.973333	0.973333
	HBEOSA-SA-NT	0.986667	0.973333	0.98
	HBEOSA-FFA	0.973333	1	0.986667
	HBEOSA-FFA-NT	0.986667	1	0.993333
	BEOSA	0.933333	0.933333	0.933333
**PenglungEW**	HBEOSA-SA	0.833333	0	0.816667
	HBEOSA-SA-NT	0.733333	0	0.766667
	HBEOSA-FFA	0.833333	0.766667	0.8
	HBEOSA-FFA-NT	0.666667	0.766667	0.716667
	BEOSA	0.933333	0.933333	0.933333
**Prostate**	HBEOSA-SA	0.952381	0.971429	0.961905
	HBEOSA-SA-NT	0.980952	1	0.990476
	HBEOSA-FFA	1	0.990476	0.995238
	HBEOSA-FFA-NT	1	1	1
**Sonar**	HBEOSA-SA	0.916667	0.785714	0.85119
	HBEOSA-SA-NT	0.892857	0.845238	0.869048
	HBEOSA-FFA	0.880952	0.785714	0.833333
	HBEOSA-FFA-NT	0.857143	0.904762	0.880952
	BEOSA	0.904762	0.904762	0.904762
**WaveformEW**	HBEOSA-SA	0.7865	0.7655	0.776
	HBEOSA-SA-NT	0.772	0.75	0.765
	HBEOSA-FFA	0.787	0.7895	0.78825
	HBEOSA-FFA-NT	0.7935	0.818	0.80575
	BEOSA	0.801	0.8	0.801
**Average**		0.910959	0.846193	0.908332

The features extracted by the hybrid binary optimizers are seen to be very competitive in terms of classification accuracy obtained on the medium-scale dataset compared to those from the high-dimensional group. A careful look at the accuracy values reported in Table 7 for experiments using 50 population size and those of 100 population size revealed that a change in population size might not have any significant performance enhancement if the hybrids of a binary optimizer are well articulated and designed. We see this confirmed in the results of Zoo, Vote, SpectEW, Lymphography, and CongressEW. Although there are some exceptions in the case of HBEOSA-SA-NT and BEOSA using Lymphography, HBEOSA-SA, and HBEOSA-FFA-NT using SpectEW, HBEOSA-SA-NT and HBEOSA-FFA using Zoo dataset, were there is a wide margin between the classification accuracies of 50 and 100 population sizes. Meanwhile, we note that the average accuracy for all the medium-scale datasets on all the hybrid methods is also significant, with the least and best being 0.783333 and 0.966667, respectively. The average classification accuracy observed for all experiments on 50 population size is 0.898019, for 100 population size is 0.866073, and the average on the individual averages is 0.904246.

**Table 7:** Medium-scale dataset comparative analysis using classification accuracy for population sizes 50 and 100.

Dataset	Algorithm	Acc50	Acc100	Avg. Acc.
**CongressEW**	HBEOSA-SA	0.95977	0.948276	0.954023
	HBEOSA-SA-NT	0.931034	0.988506	0.95977
	HBEOSA-FFA	0.971264	0.954023	0.962644
	HBEOSA-FFA-NT	0.95977	0.965517	0.962644
	BEOSA	0.942529	0.942529	0.942529
**Lymphography**	HBEOSA-SA	0.883333	0.883333	0.883333
	HBEOSA-SA-NT	0.75	0.816667	0.783333
	HBEOSA-FFA	0.85	0.85	0.85
	HBEOSA-FFA-NT	0.833333	0.883333	0.858333
	BEOSA	0.9	0	0.9
**SpectEW**	HBEOSA-SA	0.777778	0.842593	0.810185
	HBEOSA-SA-NT	0.833333	0.851852	0.842593
	HBEOSA-FFA	0.805556	0.824074	0.814815
	HBEOSA-FFA-NT	0.824074	0.907407	0.865741
	BEOSA	0.87037	0.87037	0.87037
**Vote**	HBEOSA-SA	0.908333	0.883333	0.895833
	HBEOSA-SA-NT	0.916667	0.9	0.933333
	HBEOSA-FFA	0.941667	0.92	0.933333
	HBEOSA-FFA-NT	0.95	0.983333	0.966667
	BEOSA	0.966667	0.966667	0.966667
**Zoo**	HBEOSA-SA	0.9	0.97	0.9375
	HBEOSA-SA-NT	0.9	0.8	0.875
	HBEOSA-FFA	0.925	0.8	0.8875
	HBEOSA-FFA-NT	0.95	0.9	0.95
	BEOSA	1	1	1
**Average**		0.898019	0.866073	0.904246

An interesting performance, though reduced compared to the high-dimensional dataset, on the classification accuracy is observed for the low-dimensional datasets. In Table 8, most of the accuracies obtained for the 50 and 100 population sizes are lower and ranges between [0.60–0.80]. This then motivated us to ask if those features extracted for the low-dimensional datasets were not representative of those which can yield a good classification accuracy. This concern is justified by the fact that it is desirable to have selected features produce better classification accuracy. However, since the results obtained for the low-dimensional dataset are not those for the other categories, we conclude that HBEOSA-SA selected the optimal number of features, HBEOSA-SA-NT, HBEOSA-FFA, and HBEOSA-FFA-NT, but more suggestive features were left out.

**Table 8:** Small-scale dataset comparative analysis using classification accuracy for population sizes 50 and 100.

Dataset	Algorithm	Acc50	Acc100	Avg. Acc.
**Exactly**	HBEOSA-SA	0.685	0.657	0.67125
	HBEOSA-SA-NT	0.7075	0.657	0.6825
	HBEOSA-FFA	0.7075	0.6	0.67875
	HBEOSA-FFA-NT	0.7075	0	0.70375
	BEOSA	0.98	0.9	0.98
**Exactly2**	HBEOSA-SA	0.7375	0.715	0.72625
	HBEOSA-SA-NT	0.76	0.692	0.72625
	HBEOSA-FFA	0.7525	0.7575	0.755
	HBEOSA-FFA-NT	0.7625	0.772	0.7675
	BEOSA	0.765	0.76	0.765
**Iris**	HBEOSA-SA	0.873333	0.873333	0.873333
	HBEOSA-SA-NT	0.973333	0.986667	0.98
	HBEOSA-FFA	0.96	0.946667	0.953333
	HBEOSA-FFA-NT	0.98	0.9	0.97
	BEOSA	0.966667	0.966667	0.966667
**M-of-n**	HBEOSA-SA	0.7875	0.89	0.84125
	HBEOSA-SA-NT	0.835	0.85	0.845
	HBEOSA-FFA	0.79	0.852	0.82125
	HBEOSA-FFA-NT	0.82	0.83	0.8275
	BEOSA	0.855	0.85	0.855
**Tic-tac-toe**	HBEOSA-SA	0.601563	0.739583	0.670573
	HBEOSA-SA-NT	0.726563	0.770833	0.748698
	HBEOSA-FFA	0.760417	0.700521	0.730469
	HBEOSA-FFA-NT	0.765625	0.770833	0.768229
	BEOSA	0.78125	0.7812	0.78125
**Wine**	HBEOSA-SA	0.819444	0.930556	0.875
	HBEOSA-SA-NT	0.805556	0.833333	0.819444
	HBEOSA-FFA	0.958333	0.958333	0.958333
	HBEOSA-FFA-NT	1	0.958333	0.979167
	BEOSA	0.972222	0.972222	0.972222
**Average**		0.815236	0.795755	0.819832

**Fig 4:**
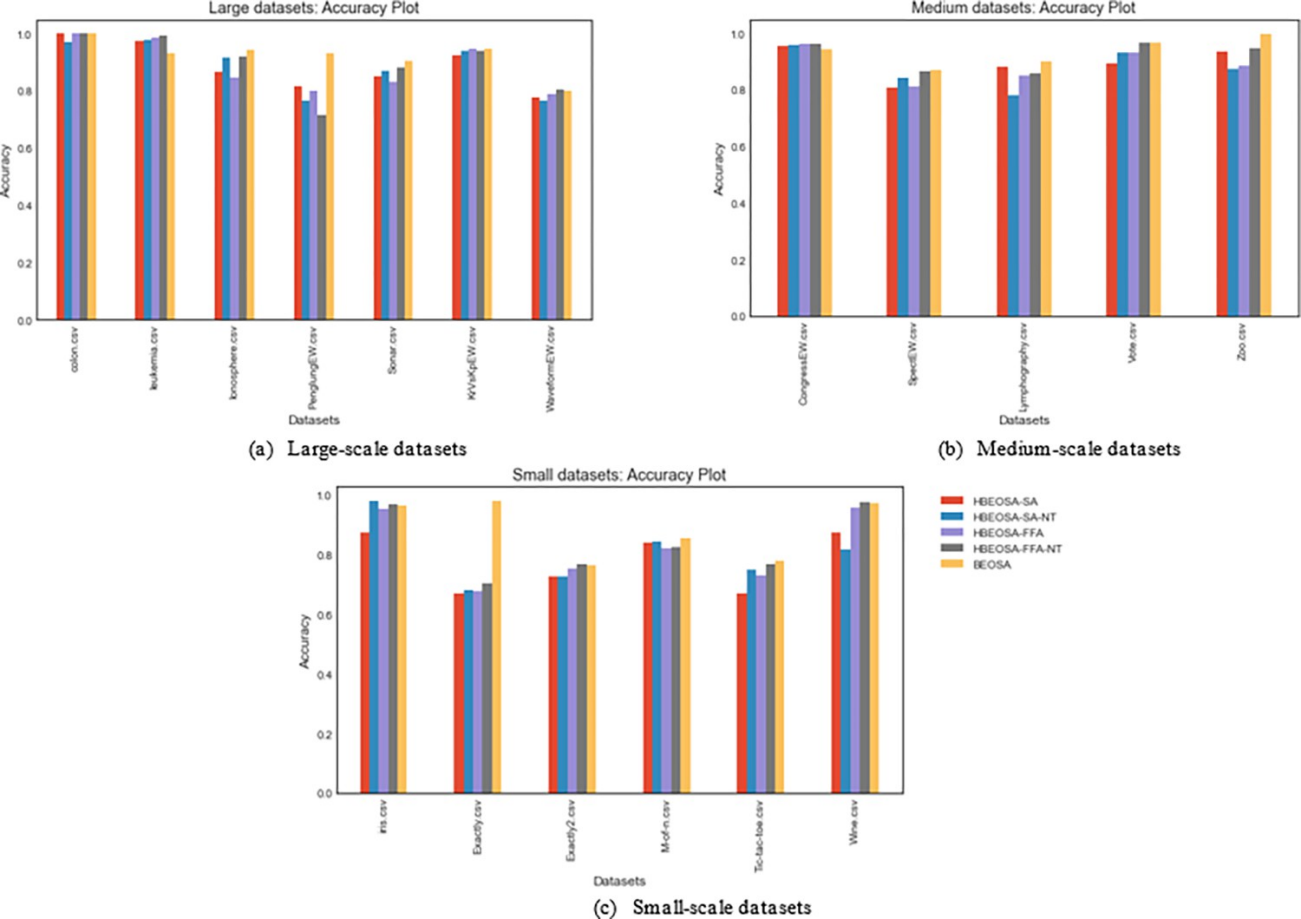
A bar chart plot for the comparison of the hybrids of BEOSA based on the classification accuracy obtained using (a) high-dimensional, (b) (medium-dimensional), and (c) low-dimensional databases.

The summary of the findings observed in the comparative analysis of the hybrid methods with respect to classification accuracy is that methods that yielded lower performance with respect to the number of features extracted still output a significant classification accuracy. Also, we noted that it is important to design binary optimizers to select the optimal number of features and include the most discriminant features capable of supporting the classifier to produce good results. To allow for having an overview of the findings from the analysis, the charts illustrating the distribution of the average classification accuracies in all the categories of datasets have been plotted, as seen in Fig 4.

In the following two sub-sections, we focus on analyzing the values returned for the fitness function, cost function, and even the computational cost for running all the hybrids compared with the single binary optimizer. This is necessary to corroborate the significance of applying the method, which yielded the impressive performance reported in the previous and this sub-sections.

### 5.3 Comparative analysis of fitness and cost values by hybrid methods

Evaluation of fitness and cost functions are very relevant to consolidating the result obtained for classification accuracy and feature counts. Whereas the fitness value demonstrates the high ranking associated with the selected solution from a wide range of candidate solutions, cost values demonstrate what is required to obtain that optimize that solution. The fitness value is expected to be minimized while the cost value is maximized, hence a min-max optimization process. In this sub-section, we analyze the fitness and cost values obtained for 50 and 100 population sizes on all categories of datasets using the hybrid methods of BEOSA.

In Table 9, the listing of the values obtained for the fitness and cost functions are outlined for population sizes 50 and 100 on all datasets listed. As observed during the discussion of the result of feature counts, we note that the fitness values of both HBEOSA-SA and HBEOSA-FFA, in some cases, returned as low as negative values. This is consistent with the feature counts reported by these same methods, where we observed that very negligent feature counts were returned, although some other cases returned positive fitness values. Again this abnormal performance is associated with using the transfer function on the hybrids, and we have already motivated the need to consider if using the transfer function in hybrids of the binary optimizer is necessary. The results obtained for HBEOSA-SA-NT and HBEOSA-FFA-NT are very impressive because, as expected, the fitness and cost values were minimized and maximized accordingly. For instance, consider the values returned for HBEOSA-SA-NT and HBEOSA-FFA-NT on WaveformEW, Sonar, PenglungEW, KrVsKpEW, Ionosphere, BreastEW, Prostate, colon, and Leukemia datasets for fitness and cost are very low and high respectively for both 50 and 100 population sizes.

**Table 9:** Large-scale dataset comparative analysis using fitness and cost values for population sizes 50 and 100.

Dataset	Algorithm	Fitnes50	Fitness100	Cost50	Cost100
**BreastEW**	HBEOSA-SA-NT	0.048088	0.038404	0.951912	0.961596
	HBEOSA-FFA	-0.13542	0.000482	1.135421	0.999518
	HBEOSA-FFA-NT	0.053439	0.07214	0.946561	0.92786
	BEOSA	0.053772	0.053772	0.946228	0.946228
**Colon**	HBEOSA-SA	-0.00019	-0.02079	1.000195	1.020793
	HBEOSA-SA-NT	0.00076	0.001704	0.99924	0.998296
	HBEOSA-FFA	-0.0344	8.503496	1.034398	0.999915
	HBEOSA-FFA-NT	0.001529	0.001239	0.998471	0.998761
	BEOSA	-0.00134	-0.00134	1.001343	1.001343
**Ionosphere**	HBEOSA-SA	0.100176	-0.73432	0.899824	1.734316
	HBEOSA-SA-NT	0.059184	0.071597	0.940816	0.928403
	HBEOSA-FFA	-0.18581	0.086622	1.185811	0.913378
	HBEOSA-FFA-NT	0.071303	0.085739	0.928697	0.914261
	BEOSA	0.05716	0.05716	0.94284	0.94284
**KrVsKpEW**	HBEOSA-SA	0.049932	-0.01482	0.949919	1.01482
	HBEOSA-SA-NT	0.082465	0.047113	0.917535	0.952887
	HBEOSA-FFA	0.047152	-0.1113	0.952848	1.111299
	HBEOSA-FFA-NT	0.06489	0.062291	0.93511	0.937709
	BEOSA	0.05471	0.05471	0.94529	0.94529
**Leukemia**	HBEOSA-SA	-0.00711	-0.23804	1.007106	1.238035
	HBEOSA-SA-NT	0.0003	0.000174	0.9997	0.999826
	HBEOSA-FFA	-0.19383	-0.01573	1.193832	1.015727
	HBEOSA-FFA-NT	0.066174	0.000207	0.933826	0.999793
	BEOSA	0.066207	0.066207	0.933793	0.933793
**PenglungEW**	HBEOSA-SA	0.0028	-0.05139	0.9972	1.051391
	HBEOSA-SA-NT	0.134338	0.001662	0.865662	0.998338
	HBEOSA-FFA	-0.04755	0.066007	1.04755	0.933993
	HBEOSA-FFA-NT	0.067692	0.070369	0.932308	0.929631
	BEOSA	0.000316	0.000316	0.999684	0.999684
**Prostate**	HBEOSA-SA	0.01683	-0.00049	0.98317	1.000486
	HBEOSA-SA-NT	0.000621	0.000597	0.999379	0.999403
	HBEOSA-FFA	-0.91025	-0.34248	1.910251	1.342475
	HBEOSA-FFA-NT	0.001193	0.000731	0.998807	0.999269
**Sonar**	HBEOSA-SA	0.073881	0.11969	0.926119	0.88031
	HBEOSA-SA-NT	0.096786	0.074381	0.903214	0.925619
	HBEOSA-FFA	-0.51461	0.023313	1.514609	0.976687
	HBEOSA-FFA-NT	0.120024	0.098952	0.879976	0.901048
	BEOSA	0.09887	0.09887	0.90113	0.90113
**WaveformEW**	HBEOSA-SA	0.201413	-1.26351	0.798568	2.263512
	HBEOSA-SA-NT	0.19484	0.19359	0.77102	0.80641
	HBEOSA-FFA	0.195719	-0.50014	0.804281	1.500139
	HBEOSA-FFA-NT	0.220345	0.191707	0.779655	0.808293
	BEOSA	0.202049	0.202049	0.797951	0.79795

The results for the medium-scale datasets are listed in Table 10 for all the hybrid methods and for population sizes 50 and 100. We noted that HBEOSA-SA, HBEOSA-FFA, HBEOSA-SA-NT, and HBEOSA-FFA-NT all performed well except for the case of HBEOSA-FFA on CongressEW (50 population size), HBEOSA-FFA on Lymphography (100 population size), HBEOSA-SA on SpectEW (50 and 100 population sizes), HBEOSA-SA and HBEOSA-FFA on Vote (50 population sizes), and HBEOSA-FFA on Zoo (50 and 100 population sizes). The fitness and cost values for both 50 and 100 population sizes on HBEOSA-SA, HBEOSA-FFA, HBEOSA-SA-NT, and HBEOSA-FFA-NT are seen to be significantly low and correspond high for Zoo, Vote, SpectEW, Lymphography, and CongressEW. The implication of this result is that the solution selected from all candidate solutions represented the best solution.

**Table 10:** Medium-scale dataset comparative analysis using fitness and cost values for population size 50 and 100.

Dataset	Algorithm	Fitness50	Fitness100	Cost50	Cost100
**CongressEW**	HBEOSA-SA	0.039138	0.027759	0.960862	0.972241
	HBEOSA-SA-NT	0.034763	0.013879	0.965237	0.986121
	HBEOSA-FFA	-0.01041	0.034111	1.010415	0.965889
	HBEOSA-FFA-NT	0.025884	0.035388	0.974116	0.964612
	BEOSA	0.060022	0.060022	0.939978	0.939978
**Lymphography**	HBEOSA-SA	0.135333	0.128501	0.864667	0.871499
	HBEOSA-SA-NT	0.104556	0.036889	0.863556	0.963111
	HBEOSA-FFA	0.104	-0.49445	0.896	1.494451
	HBEOSA-FFA-NT	0.167778	0.135333	0.832222	0.864667
	BEOSA	0.102625	0.102625	0.897375	0.897375
**SpectEW**	HBEOSA-SA	-0.00274	-0.95794	1.002743	1.957943
	HBEOSA-SA-NT	0.097415	0.077424	0.902585	0.922576
	HBEOSA-FFA	0.082253	0.122908	0.917747	0.877092
	HBEOSA-FFA-NT	0.168182	0.07993	0.831818	0.92007
	BEOSA	0.131515	0.131515	0.868485	0.868485
**Vote**	HBEOSA-SA	-0.25772	0.051375	1.257724	0.948625
	HBEOSA-SA-NT	0.002513	0.00375	0.997488	0.9962
	HBEOSA-FFA	-0.41714	0.067875	1.417141	0.93212
	HBEOSA-FFA-NT	0.051375	0.03	0.948625	0.96
	BEOSA	0.035135	0.035135	0.964865	0.964865
**Zoo**	HBEOSA-SA	0.00625	0.003125	0.89725	0.99687
	HBEOSA-SA-NT	0.05325	0.004375	0.9455	0.99562
	HBEOSA-FFA	-0.68779	-0.03985	1.687788	1.039849
	HBEOSA-FFA-NT	0.052625	0.102125	0.947375	0.89787
	BEOSA	0.005965	0.005965	0.994035	0.99403

The low-dimensional datasets Iris, Wine, Tic-tac-toe, M-of-n, HeartEW, Exactly, Exactly2, and BreastCancer were applied to the hybrid methods, and the results are listed in Table 11. The results obtained are consistent with those reported for high-dimensional and medium-dimensional categories. Some cases of the HBEOSA-SA and HBEOSA-FFA methods yield negative values for the fitness function. On the other hand, HBEOSA-SA-NT and HBEOSA-FFA-NT did well regarding the values returned for the fitness and cost functions. Note that the low-dimensional fitness results are quite high compared with those obtained for the high and medium scale datasets. Again, this points to the suitability of the proposed hybrid methods in handling high-dimensional datasets more effectively. Recall that the challenge associated with high-dimensional datasets often impairs binary optimizers’ outcomes. However, this study shows that the proposed hybrid methods are significantly suitable for high-dimensional datasets.

**Table 11:** Small-scale dataset comparative analysis using fitness and cost values for population sizes 50 and 100.

Dataset	Algorithm	Fitness50	Fitness100	Cost50	Cost100
**Exactly**	HBEOSA-SA	0.256296	0.022337	0.692331	0.977663
	HBEOSA-SA-NT	0.296665	0.307669	0.703335	0.692331
	HBEOSA-FFA	-0.10664	-0.16219	1.106644	1.162188
	HBEOSA-FFA-NT	0.301615	0.293322	0.698385	0.706678
	BEOSA	0.021638	0.021638	0.978362	0.978362
**Exactly2**	HBEOSA-SA	-0.25621	0.147335	1.256208	0.852665
	HBEOSA-SA-NT	0.238369	0.226596	0.761631	0.773404
	HBEOSA-FFA	-0.29892	0.235727	1.298919	0.764273
	HBEOSA-FFA-NT	0.235727	0.218584	0.764273	0.781416
	BEOSA	0.236496	0.236496	0.763504	0.763504
**Iris**	HBEOSA-SA	-273.018	0.150513	0.96695	1.099165
	HBEOSA-SA-NT	0.18641	0.13141	0.9595	0.964
	HBEOSA-FFA	0.03305	-0.09916	0.9975	0.901965
	HBEOSA-FFA-NT	0.0356	0.035	0.96384	0.99
	BEOSA	0.0025	0.098035	0.9645	0.964
**M-of-n**	HBEOSA-SA	0.03616	0.005	0.836985	0.832035
	HBEOSA-SA-NT	0.0355	0.035	0.866685	0.886485
	HBEOSA-FFA	0.076223	0.167965	0.908923	0.866685
	HBEOSA-FFA-NT	0.126492	0.113515	0.821838	0.818723
	BEOSA	0.091077	0.133315	0.851835	0.851835
**Tic-tac-toe**	HBEOSA-SA	0.178162	0.181277	0.764635	1.649177
	HBEOSA-SA-NT	0.148165	0.148165	0.768474	0.764898
	HBEOSA-FFA	0.235365	-0.64918	0.784554	0.783591
	HBEOSA-FFA-NT	0.231526	0.235102	0.722274	0.799102
	BEOSA	0.215446	0.216409	0.777595	0.777595
**Wine**	HBEOSA-SA	0.277726	0.200898	0.995385	1.603084
	HBEOSA-SA-NT	0.222405	0.222405	0.996154	0.970192
	HBEOSA-FFA	0.003077	-0.60308	0.996923	0.970192
	HBEOSA-FFA-NT	0.003846	0.029808	0.997692	0.940385
	BEOSA	0.003077	0.029808	0.969423	0.969423

**Fig 5:**
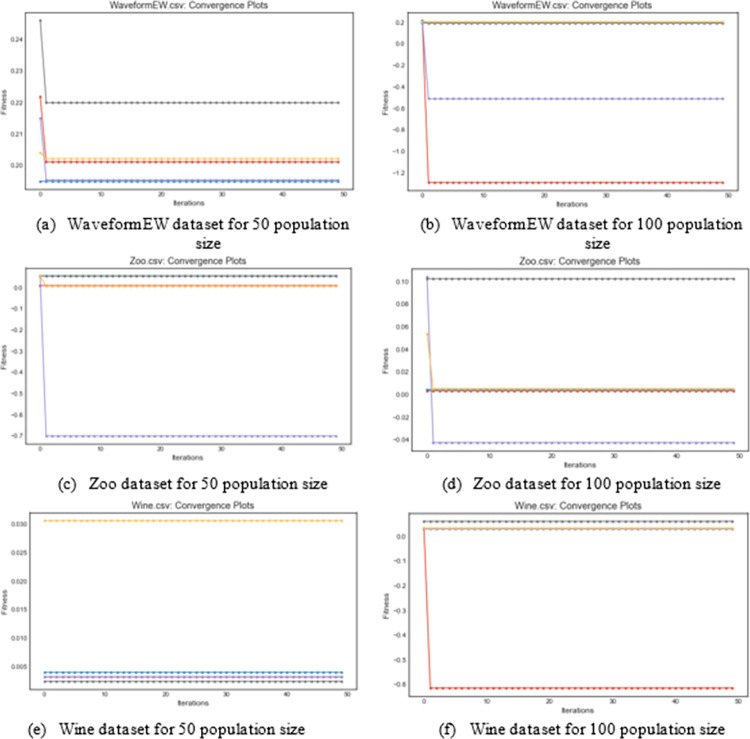
An illustration and comparison of the fitness convergence curves for the Large-scale dataset (WaveformEW), medium-scale dataset (Zoo dataset), and Small-scale (Wine dataset) using 50 and 100 population sizes. (a) WaveformEW dataset for 50 population size; (b) WaveformEW dataset for 100 population size; (c) Zoo dataset for 50 population size; (d) Zoo dataset for 100 population size; (e) Wine dataset for 50 population size; (f) Wine dataset for 100 population size.

The fitness and cost convergence curves for some selected datasets in the three categories of dataset grouping are obtained for graphing. In Fig 5, the fitness convergence curves for WaveformEW, Zoo, and Wine datasets are shown and compared with those for population sizes 50 and 100. The comparison for WaveformEW shows that the fitness curve for HBEOSA-FFA and HBEOSA-FFA-NT using 50 and 100 population sizes rank high in the plots, while those of HBEOSA-SA and HBEOSA-SA-NT trail behind. Almost the contrary is observed for the Zoo dataset belonging to medium-scale datasets. Here, both curves for HBEOSA-SA and HBEOSA-SA-NT flow high in the plots, though HBEOSA-FFA-NT successfully competes. All the hybrid methods under-performed compared with BEOSA using the Wine dataset for the 50 population size. However, we see a different curve pattern with the 100 population size plots where all hybrid methods rose high except for HBEOSA-SA. This is consistent with the report obtained from tabular data discussed earlier. Convergence curves are expected to show how the solutions benefit from the optimization process through a drop in the pattern of each curve on a plot. We see this convergence curve pattern replicated for most algorithms for each dataset except for the Wine dataset using 50 population sizes.

**Fig 6:**
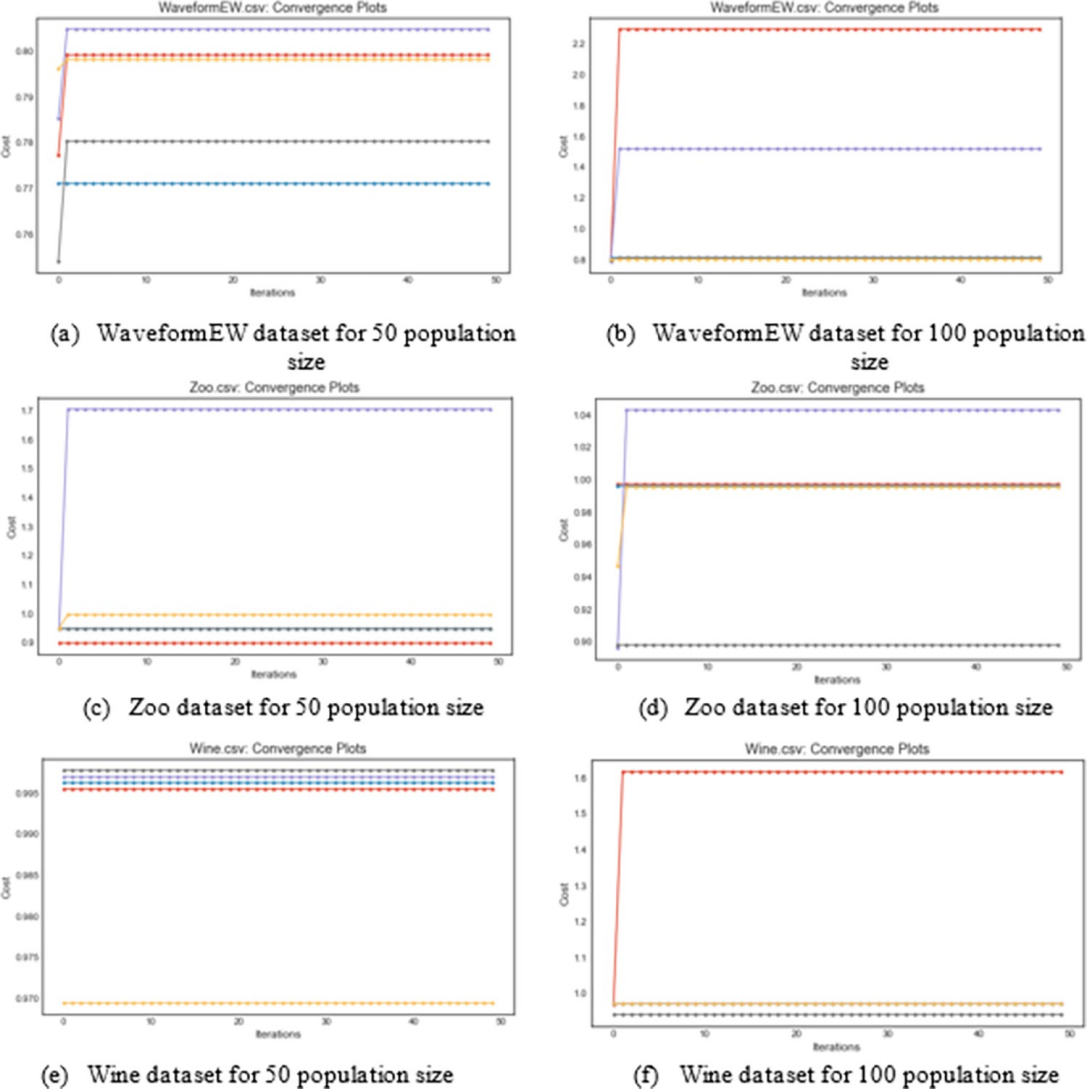
An illustration and comparison of the cost convergence curves for the Large-scale dataset (WaveformEW), medium-scale dataset (Zoo dataset), and Small-scale (Wine dataset) using 50 and 100 population sizes. (a) WaveformEW dataset for 50 population size; (b) WaveformEW dataset for 100 population size; (c) Zoo dataset for 50 population size; (d) Zoo dataset for 100 population size; (e) Wine dataset for 50 population size; (f) Wine dataset for 100 population size.

Similarly, we plot the cost function graphs for WaveformEW, Zoo, and Wine datasets for their corresponding 50 and 100 population sizes. In this case of the cost function curve, we expect each curve for the hybrid methods to rise rather than drop as defined for the fitness curves. In Fig 6, the WaveformEW dataset graph plots for 50 population size shows that HBEOSA-SA and HBEOSA-FFA rose high in the plot while those of HBEOSA-SA-NT and HBEOSA-FFA-NT were low in the plot. Almost a similar curve display is seen for the 100 population size with HBEOSA-SA at the peak, followed by HBEOSA-SA-NT, while HBEOSA-FFA and HBEOSA-FFA-NT are at the bottom of the plot. The Zoo dataset for the 50 population size shows the opposite, with HBEOSA-SA, HBEOSA-SA-NT, and HBEOSA-FFA-NT flowing at the bottom of the curve while only HBEOSA-FFA rose at the top. For the 100 population size still on the Zoo dataset, only HBEOSA-FFA-NT ranked low in the plot. All the hybrid methods performed well as graphed for the Wine dataset on the 50 population size, while only the HBEOSA-SA peaked when the population size of 100 was used. The cost curves for all the datasets on the hybrid methods are seen to rise from low points to higher points except for the Wine 50 population size.

The summary of the evaluation of the fitness and cost functions results for the low, medium, and high-dimensional datasets confirms that solutions selected best during the feature selection and classification process are indeed optimal. This is required to verify if the binary optimizers could optimize the solution space to determine the best and optimal solution out of all candidate solutions.

### 5.4 Comparative analysis of computation time by hybrid methods

Computational resources are necessary when implementing new algorithms and must be evaluated during experimentation. This study compares the computational cost of all hybrid methods considered. The discussion around this computational cost is based on the categorization of the datasets and, of course, the performance of all the hybrid methods. While we note that even the BEOSA computational cost is collected and presented, it is not used for comparison with the hybrid methods since the design approach is far different. As a result, it is observed that the computational cost of BEOSA is far lower than those of the hybrids. However, the hybrid methods achieved outstanding performance with regard to feature selection and classification. Hence, the tradeoff is to achieve improved classification accuracy using an optimal feature set at a more demanding computational cost. The following paragraphs detail the results of all methods according to their categorization in the dataset grouping.

The computational time required for running all the high-dimensional datasets is listed in Table 12. The computational cost of HBEOSA-FFA-NT is slightly higher than its corresponding HBEOSA-FFA on the BreastEW, KrVsKpEW, and Sonar datasets. However, the Prostate, Colon, Leukemia, Ionosphere, and PenglungEW datasets achieved its task at a reduced computational cost compared with HBEOSA-FFA. A comparison of the computational cost of HBEOSA-SA-NT and HBEOSA-SA on the high-dimensional datasets showed that the former is most cost-effective than the latter, as seen in Prostate, Colon, Leukemia, Sonar, and WaveformEW. Even when it appears that HBEOSA-SA recorded lower computational cost than HBEOSA-SA-NT, we noted that the difference is still insignificant. Hence, the HBEOSA-SA-NT and HBEOSA-FFA-NT are cost-effective and the most performing methods with respect to feature selection and classification. Meanwhile, all methods compete based on the computational cost listed for each high-dimensional dataset.

**Table 12:** Large-scale dataset comparative analysis using computation resources.

Dataset	Algorithm	Computation Time	Dataset	Algorithm	Computation Time
**BreastEW**	HBEOSA-SA-NT	2046.529	**Prostate**	HBEOSA-SA	4377.55
	HBEOSA-FFA	2600.879		HBEOSA-SA-NT	4000.182
	HBEOSA-FFA-NT	2700.365		HBEOSA-FFA	4539.566
	BEOSA	0.021375		HBEOSA-FFA-NT	3851.354
**Colon**	HBEOSA-SA	2501.077	**Leukemia**	HBEOSA-SA	3858.425
	HBEOSA-SA-NT	2336.011		HBEOSA-SA-NT	3040.647
	HBEOSA-FFA	2365.68		HBEOSA-FFA	3258.936
	HBEOSA-FFA-NT	2138.469		HBEOSA-FFA-NT	2749.263
	BEOSA	0.021941		BEOSA	0.015718
**Ionosphere**	HBEOSA-SA	696.8516	**PenglungEW**	HBEOSA-SA	240.0454
	HBEOSA-SA-NT	797.6596		HBEOSA-SA-NT	240.4433
	HBEOSA-FFA	728.2881		HBEOSA-FFA	246.6604
	HBEOSA-FFA-NT	996.8		HBEOSA-FFA-NT	243.8862
	BEOSA	0.012511		BEOSA	0.017666
**KrVsKpEW**	HBEOSA-SA	2911.051	**Sonar**	HBEOSA-SA	544.6124
	HBEOSA-SA-NT	2913.091		HBEOSA-SA-NT	501.1118
	HBEOSA-FFA	2763.105		HBEOSA-FFA	521.8034
	HBEOSA-FFA-NT	2831.968		HBEOSA-FFA-NT	601.6056
	BEOSA	0.012315		BEOSA	0.012638
**WaveformEW**	HBEOSA-SA	4354.99			
	HBEOSA-SA-NT	3947.937			
	HBEOSA-FFA	4293.966			
	HBEOSA-FFA-NT	4243.72			
	BEOSA	0.01185			

The computational cost of HBEOSA-SA, HBEOSA-SA-NT, HBEOSA-FFA, and HBEOSA-FFA-NT for Zoo, Vote, SpectEW, Lymphography, and CongressEW datasets are listed in Table 13. The HBEOSA-SA-NT method recorded the lowest computational cost in almost all the datasets except for the Vote dataset, where HBEOSA-FFA outperformed it. Similarly, we see the HBEOSA-SA-NT method trailing behind HBEOSA-FFA-NT in performance on low computational cost. Therefore, this implies that the removal of the transfer function on the hybrid methods produced greater benefits in terms of performance on feature selection with classification and computational cost.

**Table 13:** Medium-scale dataset comparative analysis using computation resources.

Dataset	Algorithm	Computation Time	Dataset	Algorithm	Computation Time
**CongressEW**	HBEOSA-SA	460.6276	**SpectEW**	HBEOSA-SA	631.5949
	HBEOSA-SA-NT	438.2074		HBEOSA-SA-NT	585.9336
	HBEOSA-FFA	479.9252		HBEOSA-FFA	645.2356
	HBEOSA-FFA-NT	486.5944		HBEOSA-FFA-NT	632.1654
	BEOSA	0.013038		BEOSA	0.016259
**Lymphography**	HBEOSA-SA	678.9589	**Vote**	HBEOSA-SA	498.9099
	HBEOSA-SA-NT	644.8397		HBEOSA-SA-NT	477.7804
	HBEOSA-FFA	754.0576		HBEOSA-FFA	466.5945
	HBEOSA-FFA-NT	731.7933		HBEOSA-FFA-NT	495.3158
	BEOSA	0.010652		BEOSA	0.01078
**Zoo**	HBEOSA-SA	477.6815			
	HBEOSA-SA-NT	445.674			
	HBEOSA-FFA	457.5103			
	HBEOSA-FFA-NT	482.3338			
	BEOSA	0.011132			

Table 14 reports the computational cost for the low-dimensional datasets, including Iris, Wine, Tic-tac-toe, M-of-n, HeartEW, Exactly, Exactly2, and BreastCancer. Generally speaking, a lower computational cost is reported for the low-dimensional datasets compared with the computational cost of running the algorithms on large and medium-scale datasets. This demonstrates the consistency of the hybrid algorithms and confirms their reliability and applicability to real-life optimization problems. Meanwhile, we observed that, as earlier reported, HBEOSA-SA and HBEOSA-FFA’s computational cost was lower than their corresponding models, HBEOSA-SA-NT and HBEOSA-FFA-NT, which do not use transfer functions. For instance, the computational cost for HBEOSA-SA and HBEOSA-FFA using the Exactly, M-of-n, Tic-tac-toe (853.813 and 902.6218, 801.7767 and 801.7767, 1599.557 and 1551.322, 832.3618 and 946.9568) and Wine are as against those of HBEOSA-SA-NT and HBEOSA-FFA-NT (933.5138 and 1122.875, 893.3054 and 800.887, 1793.579 and 1702.181, 797.7159 and 871.5879) respectively. However, for the Exactly2 and Iris datasets, performance for HBEOSA-FFA on computational cost was higher than its corresponding HBEOSA-FFA-NT.

**Table 14:** Small-scale dataset comparative analysis using computation resources.

Dataset	Algorithm	Computation Time	Dataset	Algorithm	Computation Time
**Exactly**	HBEOSA-SA	853.8136	**M-of-n**	HBEOSA-SA	801.7767
	HBEOSA-SA-NT	933.5138		HBEOSA-SA-NT	893.3054
	HBEOSA-FFA	902.6218		HBEOSA-FFA	846.8971
	HBEOSA-FFA-NT	1122.875		HBEOSA-FFA-NT	800.887
	BEOSA	0.010938		BEOSA	0.010155
**Exactly2**	HBEOSA-SA	952.5927	**Tic-tac-toe**	HBEOSA-SA	1599.557
	HBEOSA-SA-NT	1028.579		HBEOSA-SA-NT	1793.579
	HBEOSA-FFA	1035.1		HBEOSA-FFA	1551.322
	HBEOSA-FFA-NT	920.2004		HBEOSA-FFA-NT	1702.181
	BEOSA	0.010504		BEOSA	0.014264
**Iris**	HBEOSA-SA	2051.599	**Wine**	HBEOSA-SA	832.3618
	HBEOSA-SA-NT	2008.141		HBEOSA-SA-NT	797.7159
	HBEOSA-FFA	1967.045		HBEOSA-FFA	946.9568
	HBEOSA-FFA-NT	1825.945		HBEOSA-FFA-NT	871.5879
	BEOSA	0.015686		BEOSA	0.010729

**Fig 7:**
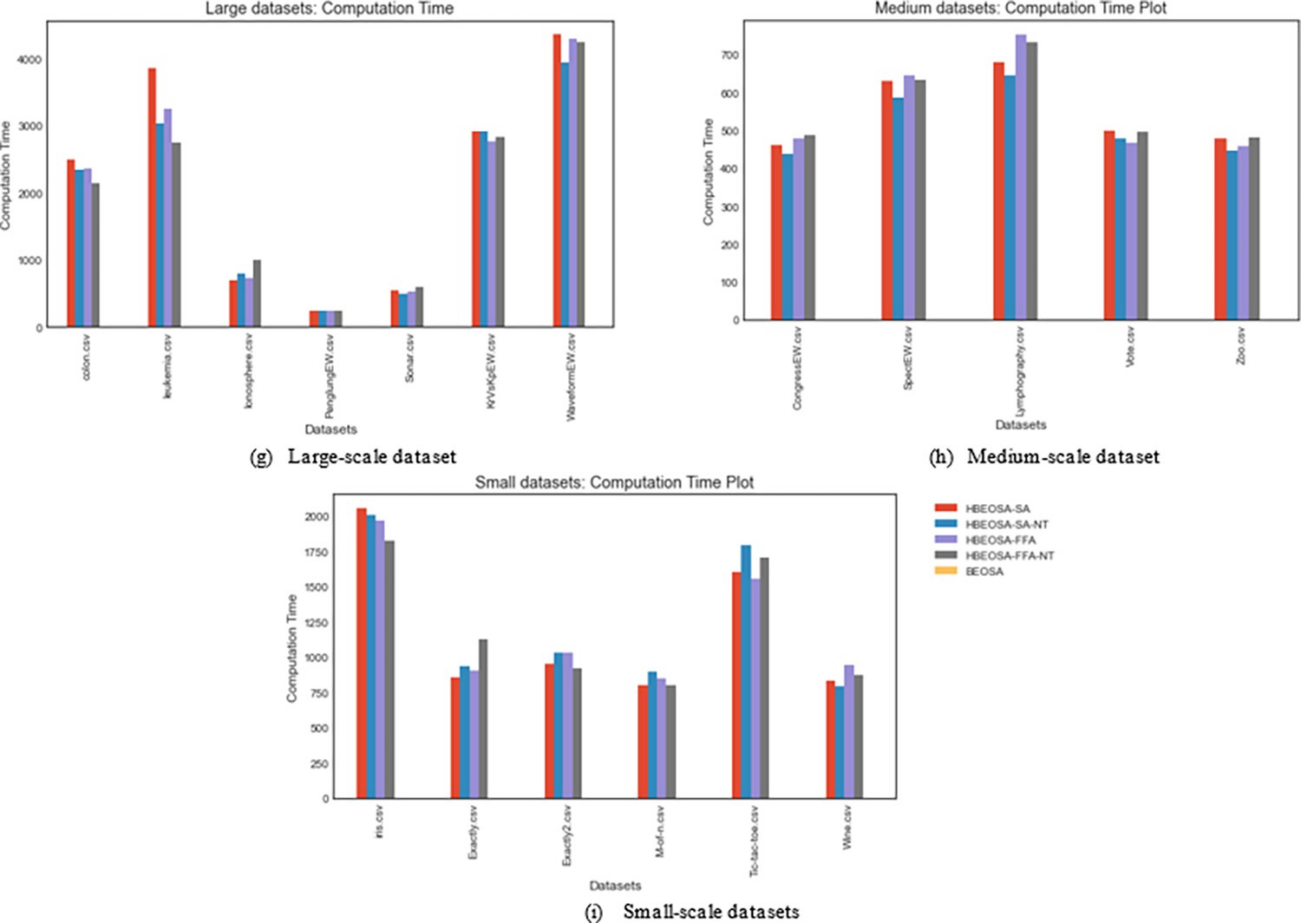
A bar chart plot for the comparison of the hybrids of BEOSA, namely HBEOSA-SA, HBEOSA-SA-NT, HBEOSA-FFA, HBEOSA-FFA-NT based on the computational resource obtained using (a) high-dimensional, (b) (medium-dimensional) and (c) low-dimensional databases.

The computational cost discussed in previous paragraphs for the three categories of datasets is further presented using graphs for clarification. In Fig 7, we apply bar charts to show the distribution of computational cost for each hybrid algorithm. The high-dimensional datasets, Sonar, PenglungEW, and Ionosphere are computationally low compared with those of WaveformEW, KrVsKpEW, BreastEW, Prostate, Colon, and Leukemia. In almost all the datasets, the bar column for HBEOSA-SA is seen to peak higher above other methods. This is contrary to what is seen with the medium and low dimensional datasets, where the bar columns for HBEOSA-FFA and HBEOSA-SA-NT, respectively, were higher in computational plotting than the other hybrid methods.

The summary of the computational cost observed for all the hybrid methods for the three categories of datasets showed that this resource cost is justified by the gain achieved on the reduced feature selected and the classification accuracy. We note that this corroborates with the study’s aim, which seeks to promote a hybrid binary optimizer that outperforms a single binary optimizer at a considerable computational cost.

### 5.5 Discussion on findings

In this sub-section, the findings from the study are presented through a combinatorial observation of the performance of all hybrid algorithms on fitness, classification accuracy, and cost. Recall that we have noted that based on individual approaches for examining these metrics, we confirmed that the results obtained were consistent with the features selected by each method. However, to arrive at justifiable findings, we applied radar plots to chart these three metrics on a single graph for some selected datasets in each dataset category.

**Fig 8:**
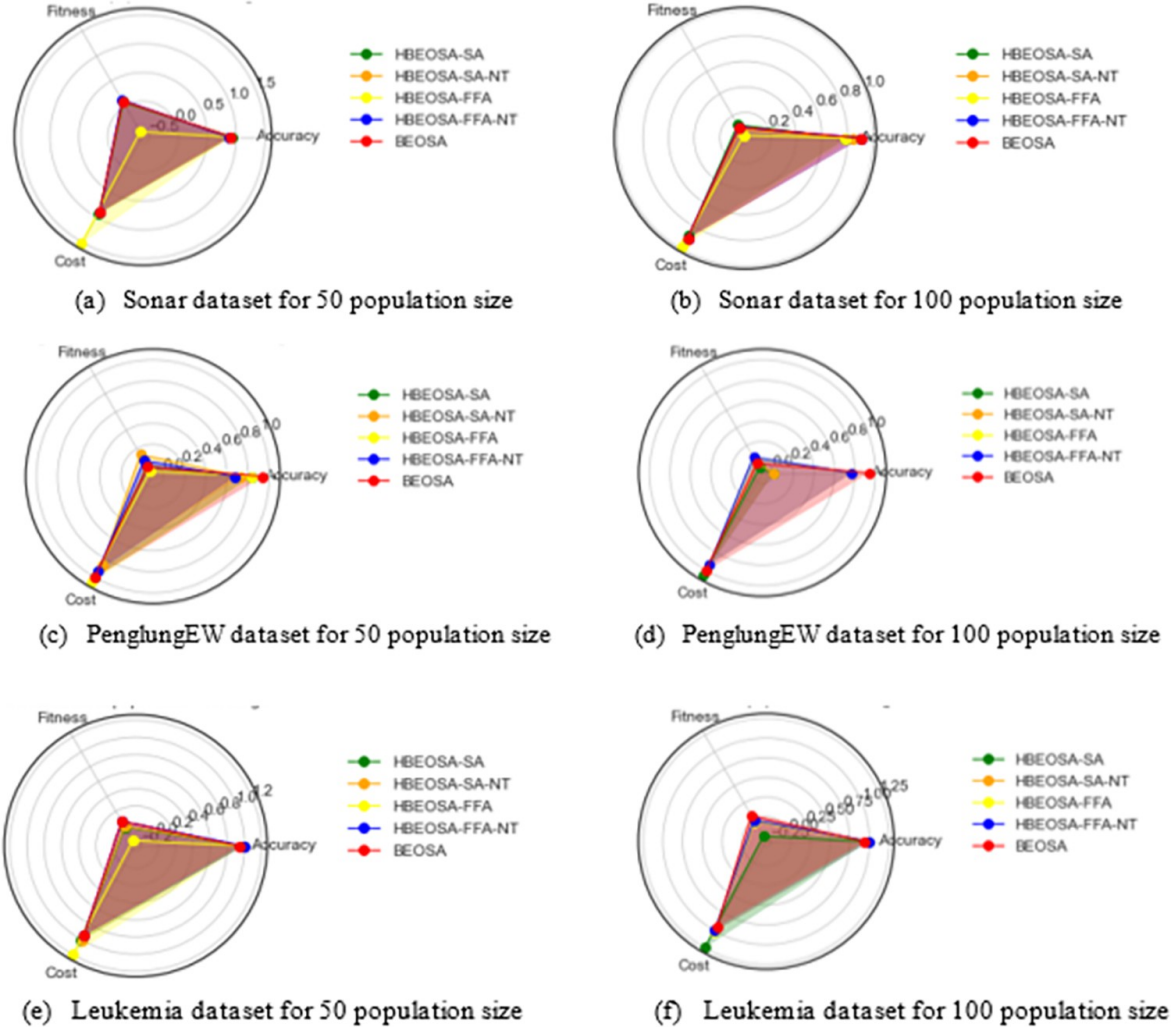
A radar plot illustrating the comparison of the classification accuracy, cost values, and fitness values for the hybrids of BEOSA when applied to some high-dimensional datasets using a variation of 50 and 100 population sizes. (a) Sonar dataset for 50 population size; (b) Sonar dataset for 100 population size; (c) PenglungEW dataset for 50 population size; (d) PenglungEW dataset for 100 population size; (e) Leukemia dataset for 50 population size; (f) Leukemia dataset for 100 population size.

In Fig 8, we selected the fitness, classification accuracy, and cost values on Sonar, PenglungEW and Leukemia datasets for 50 and 100 population sizes and plotted them using radar plot. Again, placing graphs for both 50 and 100 population sizes close will buttress the findings further if considerable population sizes influence the performance of hybrids methods. For the Sonar dataset, we noted a strong alignment of values returned for fitness, accuracy, and cost for HBEOSA-SA, HBEOSA-SA-NT, and HBEOSA-FFA-NT in both the 50 and 100 population sizes, the exception to this is the plot for the HBEOSA-FFA algorithm. The values for fitness, accuracy, and cost using the PenglungEW dataset, the lag existing among plots for the hybrid methods is very small on both the 50 and 100 population sizes. On the Leukemia dataset, the small lag in plots exists only for the fitness, accuracy, and cost values with the HBEOSA-FFA algorithm using 50 population size and values for fitness, accuracy, and cost with the HBEOSA-SA algorithm using 100 population size. This shows that for all the hybrid methods proposed in the study, there is a correlation in the fitness performance, accuracy, and cost relating to the feature selected. This implies that when the performance for fitness, accuracy, and cost are poor, the aim of the hybrid methods in minimizing the number of features selected will be defeated. This demonstrates the need to consider the performance of binary optimizers not only by examining metrics on an individual basis but rather by correlating the values from related metrics in a manner that will project the harmonious behavior of the optimizer in executing the feature selection task. Secondly, the study’s findings showed minimal performance gain when the population size varied for each hybrid method. In fact, we noted that even the single binary optimizer appears to trail behind its corresponding hybrids in terms of performance. A confirmation that hybrid binary optimizers will maintain the behavioral pattern of their corresponding single/base method while improving performance. Also, as charted in the plots, results showed that the proposed hybrid methods are very suitable for addressing high-dimensional datasets with no abnormality observed.

**Fig 9:**
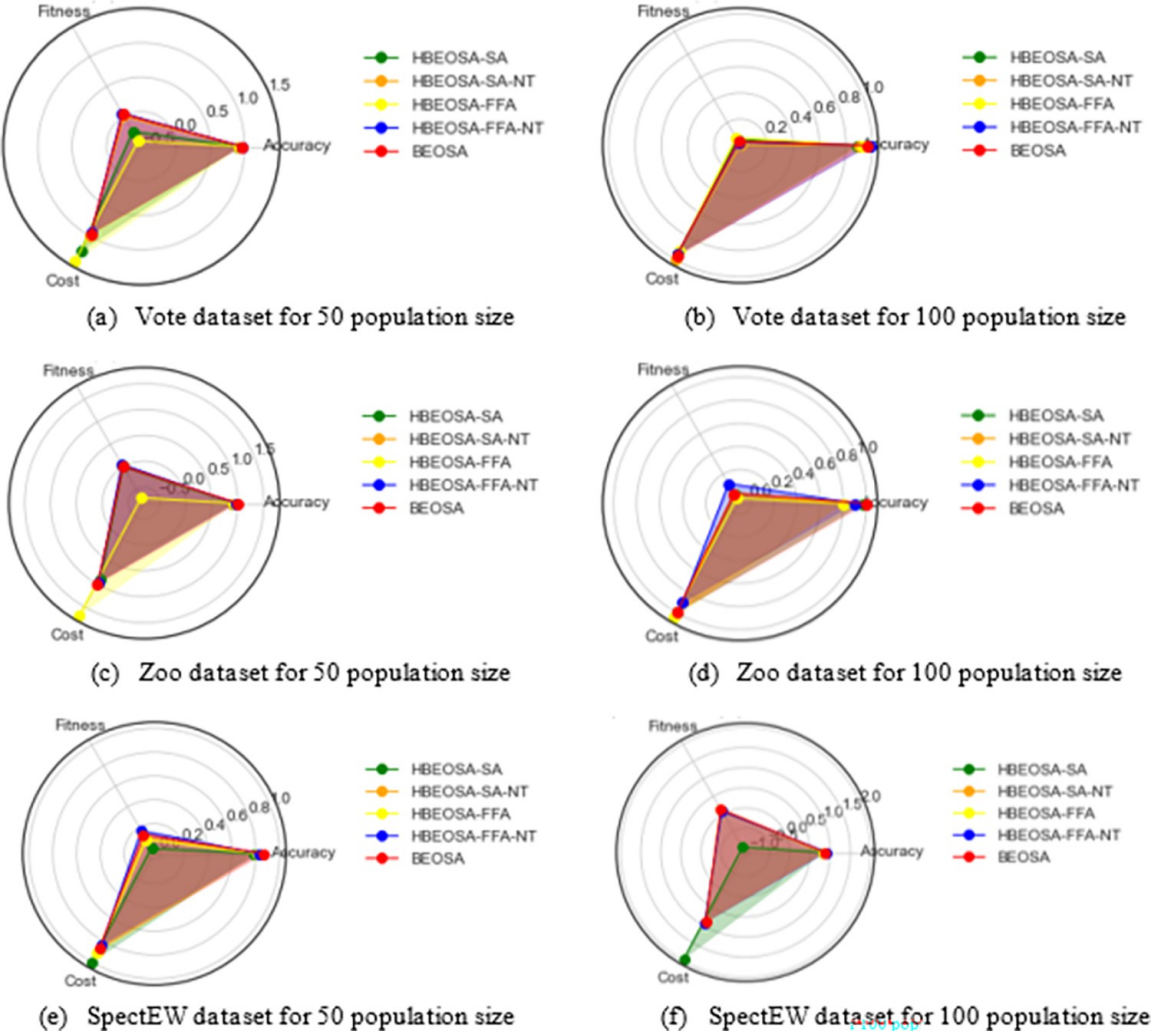
A radar plot illustrating the comparison of the classification accuracy, cost values, and fitness values for the hybrids of BEOSA when applied to some medium-sized dimensional datasets using a variation of 50 and 100 population sizes. (a) Vote dataset for 50 population size; (b) Vote dataset for 100 population size; (c) Zoo dataset for 50 population size; (d) Zoo dataset for 100 population size; (e) SpectEW dataset for 50 population size; (f) SpectEW dataset for 100 population size.

Further investigation on the behavior of the hybrid methods is observed for both the medium-scale datasets are reported in Fig 9. The Vote, Zoo, and SpectEW datasets were randomly selected for analyzing the performance of the medium-scale datasets using the 50 and 100 population sizes. On the Vote dataset, the fitness and cost values for the hybrid methods using a 50 population size were better than the single BOESA method. Classification accuracies lapped for all hybrid and single methods. This lap is also noticed with the 100 population size. Using the Zoo dataset, we see a repletion of this lap of the plots for the hybrid and the single binary methods, except for the HBEOSA-FFA, which reported a more desirable result using the 50 population size. Also, this competitive performance for the fitness, cost, and classification accuracy for SpectEW is demonstrated through the lap in the plots between the hybrid methods and the single binary optimizer method. This shows that the hybrid binary optimizer performs almost similar to the single binary optimizer with the medium-scale dataset. This then shows that large-scale dataset stands to benefit more from the concept of hybridization of single binary optimizers. Moreover, we see that even with the medium-scale dataset, the hybrid methods performed well only that there was no significant difference in their performance compared with the single binary optimizer.

**Fig 10:**
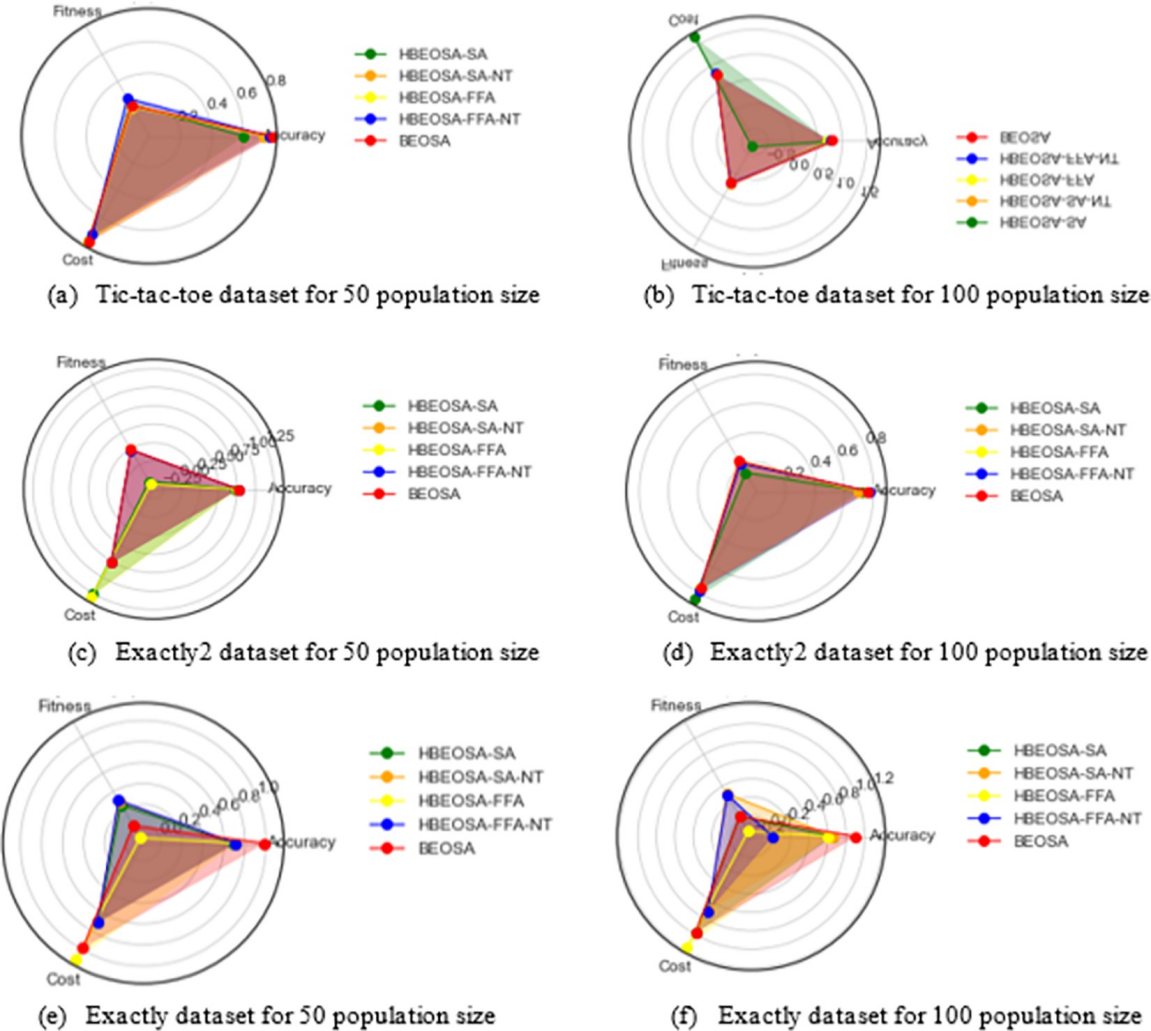
A radar plot illustrating the comparison of the classification accuracy, cost values, and fitness values for the hybrids of BEOSA when applied to some small-sized dimensional datasets using a variation of 50 and 100 population sizes. (a) Tic-tac-toe dataset for 50 population size; (b) Tic-tac-toe dataset for 100 population size; (c) Exactly 2 dataset for 50 population size; (d) Exactly 2 dataset for 100 population size; (e) Exactly dataset for 50 population size; (f) Exactly dataset for 100 population size.

The findings from applying the low-dimensional datasets to the proposed hybrid methods are illustrated using the plots in Fig 10. The Tic-tac-toe, Exactly2, and Exactly datasets were randomly selected from this category for the comparative analysis of fitness, cost, and classification accuracy values. With the Tic-tac-toe dataset using a 50 population size, we see no significant difference between the hybrid methods and the single binary optimizer. However, for the experiment using a 100 population size, only the HBEOSA-SA benefited more with respect to fitness and cost, while all the other hybrid algorithms overlap in performance. A similar pattern is observed for the Exactly2 dataset, especially when using the 100 population size. However, that which uses a 50 population size revealed better performance for HBEOSA-SA and HBEOSA-FFA in terms of fitness and cost values. On the contrary, both HBEOSA-SA and HBEOSA-FFA-NT reported a slight drop in performance on fitness and cost values when 50 population size is used, and HBEOSA-SA-NT and HBEOSA-FFA-NT a similar drop in performance when 100 population size is used on the Exactly dataset. The remaining two hybrid algorithms outperformed the single binary optimizer in both cases.

Recall that the motivation for this study is to investigate the possible performance enhancement when nested transfer functions are applied to solve the FS problem, as against the traditional single-function approach. We also noted that the study aims to observe the performance gain of using the threshold method compared with the transfer function method in binarizing the continuous optimizer process. The outcome of the study has shown that the following observations were the reason for the results obtained:

- It is expected that there must exist a correlation between the values returned for fitness, accuracy, and cost functions. We observed that in most cases for the hybrid algorithms, this correlation holds to buttress that all results and performance enhancement achieved make the hybrid algorithms valid and relevant in solving the FS problem. Furthermore, we noted that values obtained for these three functions (fitness, accuracy, and cost) were, in most cases, demonstrating a form of alignment even when the population size varied between 50 and 100. This is readily noticeable with HBEOSA-SA, HBEOSA-SA-NT, and HBEOSA-FFA-NT algorithms, most of which are the methods using the nested-transfer functions. The reason for this performance is to confirm the use of the nested-transfer function as a stabilizer of binary optimizers even when solving FS problems using high-dimensional datasets. This is indeed very impressive considering the problematic nature of high-dimensional datasets with use on binary optimizers.
- Another observation noted with the results obtained during the experimentation process using a particular population size, 50, revealed no significant difference between the hybrid methods and the single binary optimizer. Again, the nested transfer function justifies this performance since it stabilizes the candidate solutions.
- When using low dimensional datasets, an observation noted for the fitness and cost values results confirmed that the hybrid optimizers were more optimal in performance than the single-optimizer. The reason for this is based on the mutual benefit derived from leveraging the composing algorithms’ strengths. As typical of previous observations, the hybrid methods using nested-transfer functions are leading in this respect.

The summary of the findings from the study on the use of hybrid binary optimizers is that such methods improve performance in addressing feature selection problems compared with their corresponding single binary optimizers. This performance enhancement is seen to be reflected in the quantity and quality of features selected and the fitness and cost of the best solution selected from candidate solutions. Meanwhile, competitive performance is observed between the hybrid methods and the single binary optimizers when both the medium-scale and low-dimensional datasets are used. These findings imply that high-dimensional datasets benefit more from a hybrid binary optimizer than a single binary optimizer. Moreover, recall that the computational cost for hybrid binary optimizers is much higher than those for single binary optimizers. Therefore, this study’s findings show significant performance gain for the high-dimensional dataset, which is an interesting discovery. This is because most real-life problems are characterized by high-dimensional datasets, which are highly solvable with better performance using the proposed hybrid binary optimizers.

## 6. Conclusion

The use of hybrid binary optimization algorithms is proposed and investigated in this study. For the single binary optimizer, the binary Ebola optimization search algorithm (BEOSA) is used as the basis for deriving the hybrid algorithms. In designing the hybrid methods, the simulated annealing (SA) and the firefly algorithm (FFA) were hybridized with BEOSA to achieve both HBEOSA-SA and HBOESA-FFA. A further investigative study on the influence of transfer functions in the design of hybrid methods was conducted. Results showed that the hybrid algorithms not designed to use transfer functions outperformed those which used the functions. Findings from the study also showed that studies on binary optimization algorithms need to consider the performance of binary optimizers not only by examining metrics on an individual basis but by correlating the values from related metrics in a manner that will project harmonious behavior of the optimizer. The study also investigated the influence of increasing population sizes of the solutions in the search space. The result confirmed that there is minimal performance gain when the population size is varied for each hybrid method. Furthermore, the hybrid algorithms reported performances almost similar in pattern to those of the single binary optimizer. This showed that hybrid binary optimizers would maintain the behavioral pattern of their corresponding single/base method while improving performance. The datasets applied for the experimentation were categorized into high-dimensional, low-dimensional, and medium-scale dimensions. The experiment’s outcome revealed that the hybrid methods performed better than those datasets in the other two categories. This then shows that large-scale dataset stands to benefit more from the concept of hybridization of single binary optimizers. In future work, we recommend investigating other transfer functions on the hybrid methods to investigate the possible performance behavior. Meanwhile, the use of the threshold method other than the transfer function method can be investigated to draw a comparative analysis of their performance. Recent discrete and continuous optimizers might as well be considered for hybridization with the BEOSA method to reveal further how efficiently the new hybrid methods might perform compared with what is reported in this study.
